# WWP1 upregulation predicts poor prognosis and promotes tumor progression by regulating ubiquitination of NDFIP1 in intrahepatic cholangiocarcinoma

**DOI:** 10.1038/s41420-022-00882-0

**Published:** 2022-03-09

**Authors:** Yongjian Li, Qian Cheng, Jie Gao, Zhuomiaoyu Chen, Jingheng Guo, Zuyin Li, Lingyu Tian, Chao Zhang, Yuzi Li, Jiaxi Zheng, Zhao Li, Jiye Zhu

**Affiliations:** 1 Department of Hepatobiliary Surgery, Peking University People’s Hospital, Beijing, China; 2 Beijing Key Laboratory of HCC and Liver Cirrhosis, Peking University People’s Hospital, Beijing, China; 3 Peking University Center of Liver Cancer Diagnosis and Treatment, Peking University People’s Hospital, Beijing, China; 4 Peking University Institute of Organ Transplantation, Peking University People’s Hospital, Beijing, China

**Keywords:** Bile duct cancer, Oncogenes, Hepatocellular carcinoma, Mechanisms of disease, Ubiquitylation

## Abstract

WW domain-containing E3 ubiquitin protein ligase1 (WWP1) is reported to be upregulated in many types of human cancers; however, its expression and function in intrahepatic cholangiocarcinoma (ICC) remain unknown. Here, in this study we investigated the expression pattern, clinical prognosis, tumor biological functions, and molecular mechanisms of WWP1 in ICC. The expression of WWP1 in patient tissues was detected by western blotting, immunohistochemistry (IHC), and immunofluorescence. CCK-8, colony formation, EdU, transwell, and xenograft models were used to explore the role of WWP1 in the proliferation and metastasis of ICC. Co-immunoprecipitation, mass spectrometry, chromatin immunoprecipitation, and immunofluorescence were performed to detect the potential mechanisms. Our study revealed that WWP1 was highly expressed in ICC, and high levels of WWP1 were associated with poor prognosis. Functionally, WWP1 overexpression enhanced the proliferation and metastasis of ICC cells and vice versa. Mechanistically, MYC could be enriched in the promoter region of WWP1 to facilitate its expression. Then, WWP1 targets Nedd4 family interacting protein1 (NDFIP1) and reduces NDFIP1 protein levels via ubiquitination. Downregulation of NDFIP1 in ICC cells rescued the effects of silenced WWP1 expression. WWP1 expression was also negatively correlated with the protein level of NDFIP1 in patient tissues. In conclusion, WWP1 upregulated by MYC promotes the progression of ICC via ubiquitination of NDFIP1, which reveals that WWP1 might be a potential therapeutic target for ICC.

## Introduction

Intrahepatic cholangiocarcinoma (ICC) is a type of cholangiocarcinoma located proximally to the second-degree bile ducts [1]. With increasing incidence, ICC is the second most common malignancy of the liver after hepatocellular carcinoma (HCC) [1–3]. Owing to its high aggressiveness and invasive malignancy, the diagnosis and treatment are very complicated resulting in poor outcomes. Recent studies have shown that ICC is mainly derived from epithelial cells of the biliary tract in the liver. In the process of tumorigenesis, some genetic changes have been identified, such as IDH1/2, FGFRs, EGFRs, KRAS, and BRAF [1], which lead to the dysregulation of some fetal cell survival pathways, such as RAS-RAF-MEK-ERK signaling or PI3K-AKT-mTOR signaling [4]. Although some mechanisms underlying the carcinogenesis of ICC have been established, there is limited information on the efficacy of therapy targeting this disease. Therefore, efforts focused on discovering new molecular targets of ICC are necessary.

WW domain-containing E3 ubiquitin protein ligase1 (WWP1), which belongs to the NEDD4-like family, plays multiple functions such as cell proliferation, senescence, protein trafficking, transcription, and regulation of epithelial sodium channels [5, 6]. The human *WWP1* gene is located on chromosome 8q21, and the WWP1 protein is composed of three main parts: an N-terminal C_2_ domain, four WW domains, and a C-terminal catalytic HECT domain [7]. The function of the WW domains is to mediate interactions between WWP1 and other proteins by recognizing PPxY motifs [8, 9]. Recently, many studies revealed that the abnormal regulation of WWP1 participated in many diseases, such as, neurological disorders [10], dystroglycan-related disorders [11], cardiac hypertrophy [12], and cancers [7, 13]. Interestingly, Novelli et al. [14] reported that high-expression WWP1 in the lung tissues of COVID-19 patients heralded more serious COVID-19 symptoms.

Accumulating evidence demonstrates that WWP1 is aberrantly expressed in various types of malignancies, indicating that WWP1 serves as an oncoprotein or a tumor suppressor [7, 15]. For instance, high expression of WWP1 promotes cell growth of prostate tumor cells by mediating the K27-linked PTEN polyubiquitination [16]. Moreover, WWP1 regulates the malignant activity of breast cancer by specifically ubiquitinating the ErbB4 protein [17]. Nevertheless, the role of WWP1 in ICC carcinogenesis and progression of ICC remains unknown.

Nedd4 family interacting protein1 (NDFIP1), serves as an adaptor of Nedd4 family proteins, in which PPxY motifs of NDFIP1 directly bind to the WW domains of Nedd4 family proteins [18, 19]. Experimental studies have revealed that NDFIP1 has potential anti-tumor effects in multiple malignant diseases. For instance, miR-873 inhibits the expression of NDFIP1 to promote the progression of hepatocellular carcinoma [20]. However, the role and related mechanisms of NDFIP1 in ICC are not well defined. The purpose of the current study was to investigate the expression pattern of WWP1 in ICC and to explore the related mechanisms

In this study, we revealed that WWP1 is overexpressed in ICC and serves as a function of tumorigenesis. We confirmed that MYC could enrich the promoter of the WWP1 gene to enhance its expression. Moreover, we found that WWP1 interacts with NDFIP1. In addition, we found that WWP1 could reduce the protein level of NDFIP1 via ubiquitination. Generally, this study sheds new light on the tumorigenesis of ICC, providing a potential therapeutic strategy for patients with ICC.

## Results

### WWP1 was upregulated in tumor tissues and its high expression indicated a poor prognosis in ICC

**Fig. 1:**
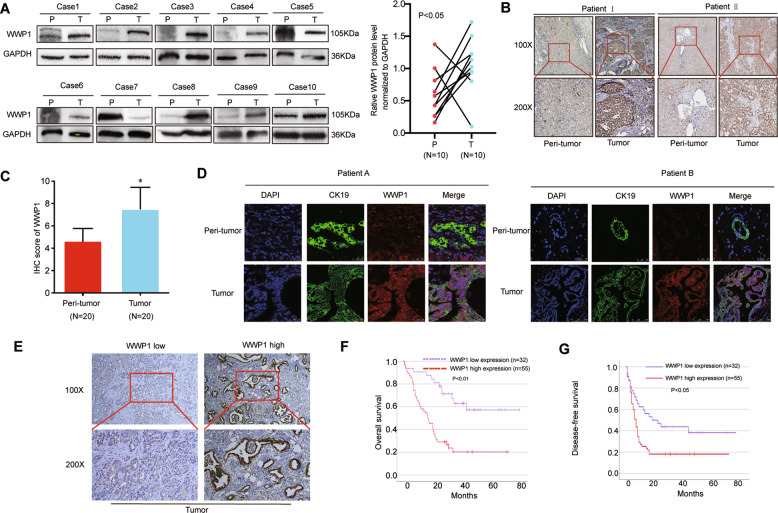
WWP1 was upregulated in ICC and its high expression was related to poor prognosis. **A** Images of WWP1 protein levels in ICC tumor and paired adjacent non-tumor tissues by western blotting assays. P peritumoral tissues, T tumor tissue, *n* = 10. **B** Representative images of WWP1 protein levels in paraffin-embedded specimens performed by IHC staining. Magnifications, ×100 and ×200, *n* = 20. **C** The scores of WWP1 staining intensity of IHC. **D** Images of WWP1 expression levels in CK19-positive tumor cells and CK19-positive normal epithelial cells of adjacent non-tumor liver tissues by immunofluorescence staining (blue: DAPI; green: CK19; red: WWP1). **E** Representative IHC images of low WWP1 expression and high WWP1 expression in ICC tumor tissues. **F**, **G** Kaplan-Meier survival analyses were performed to detect the effect of WWP1 on overall survival (**F**) or disease-free survival (**G**). *P* is based on the log-rank test. ICC intrahepatic cholangiocarcinoma, IHC immunohistochemistry. *P < 0.05.

First, we explored the expression patterns of WWP1 in ICC between tumor tissues and paired adjacent normal liver tissues. We randomly selected the samples from conformed ICC patients who underwent surgery at our hospital in the past 2 years and performed western blotting analysis and immunohistochemistry (IHC) analysis. The results of western blotting showed that 80% (8/10) of tumors displayed significantly higher expression of WWP1 compared to paired adjacent normal liver tissues (Fig. 1A). The IHC results revealed that the staining score of WWP1 in the ICC tumor tissues was higher than that in the paired adjacent non-tumor tissues (Fig. 1B). Because ICC mainly originated from normal epithelial cells of biliary tracts in the liver, we further investigated WWP1 expression in the other three ICC samples and the paired normal epithelial cells of intrahepatic bile ducts by double immunofluorescence (IF) assay. Double IF staining of CK19 and WWP1 showed that the expression of WWP1 was upregulated in CK19-positive tumor cells compared with that in CK19-positive epithelial cells of adjacent non-tumor tissues (Fig. 1C and Fig. S1A).

To further investigate whether WWP1 expression was associated with ICC prognosis, ICC samples from a cohort of 87 patients who underwent curative resection in our hospital were immunostained. WWP1 expression was divided into high and low expression groups as seen in Fig 1E. The relationship between WWP1 expression and patient clinicopathological features is shown in Table 1. Kaplan–Meier analysis revealed that ICC samples with high WWP1 expression had shorter overall survival (OS) (Fig. 1F) and higher recurrence (Fig. 1G) than ICC samples with low WWP1 expression (25.36 vs. 54.99%, OS; 17.74 vs. 35.92%, DFS; both *P* < 0.05). All evidence suggests that WWP1 might be a potential risk factor for patients with ICC.

**Table 1:** Correlation between WWP1 and clinicopathological characteristics in ICC patients.

Variable	WWP1 expression		*P* value
	Low (*n* = 32)	High (*n* = 55)	
Sex (%)			
Female	19 (59.4)	21 (38.2)	0.09
Male	13 (40.6)	34 (61.8)	
Age, years	59.1 ± 11.6	60.6 ± 10.2	0.54
HBsAg (%)			
No	13 (40.6)	33 (60.0)	0.08
Yes	19 (59.4)	22 (40.0)	
CA19-9 (UI/mL)	318.9 ± 407.6	308.1 ± 408.6	0.91
CEA (UI/mL)	20.7 ± 64.3	12 ± 37.6	0.43
Cirrhosis (%)			
No	25 (78.1)	45 (81.8)	0.07
Yes	7 (21.9)	10 (18.2)	
Tumor diameter, cm	6.4 ± 3.6	5.2 ± 2.9	0.1
MVI (%)			
No	21 (65.6)	44 (80)	0.22
Yes	11 (34.4)	11 (20)	
TNM (%)			
I	11 (34.4)	19 (34.5)	0.98
II	6 (18.8)	11 (20)	
III	15 (46.9)	25 (45.5)	

### WWP1 promoted the ICC cells proliferation, migration, and invasion

**Fig. 2:**
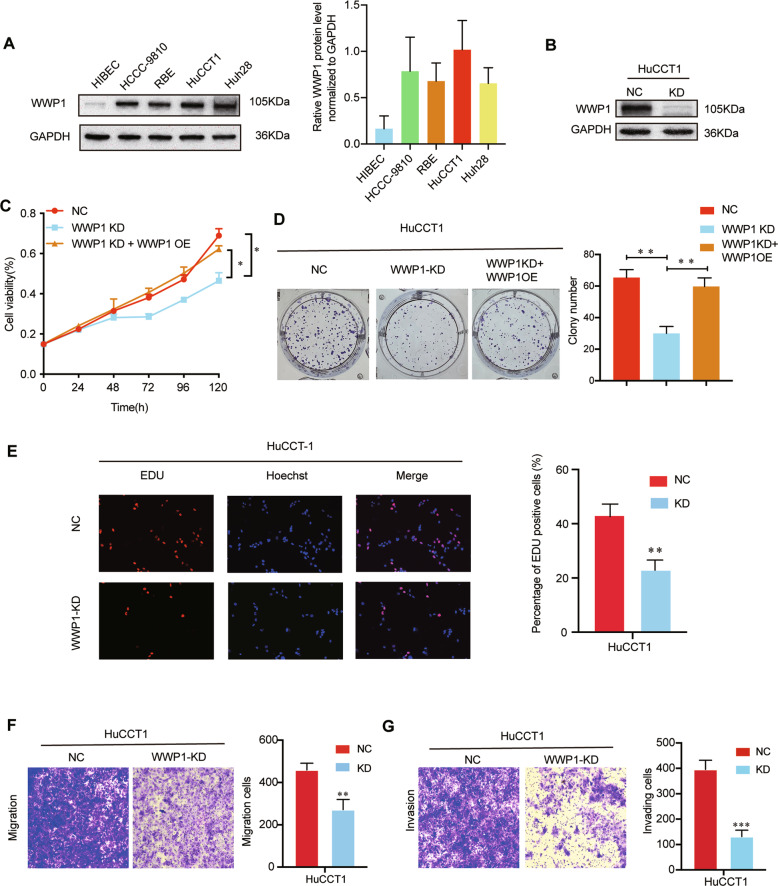
WWP1 silencing suppressed proliferation, migration, and invasion of HuCCT1 cells in vitro. **A** WWP1 protein levels in the four ICC cell lines and normal human intrahepatic biliary epithelial cell (HIBEC) were determined by western blotting. **B** The efficiency of WWP1 knockdown was estimated by western blotting. **C** CCK-8 assay, **D** colony formation assay, **E** EdU assay were performed to estimate the proliferation of HuCCT1 cells after WWP1 silencing, *n* = 3. Overexpression of WWP1 again in HuCCT1 cells infected with the lentiviral shWWP1 rescued the ability of proliferation (**C**, **D**). **F**, **G** Transwell assays were performed to detect the migration (**F**) and invasion (**G**) of HuCCT1 cells after WWP1 silencing, *n* = 3. **P < 0.01, ***P < 0.001.

The western blotting assay showed that the WWP1 protein level of the normal human intrahepatic biliary epithelial cell (HIBEC) was significantly lower than that of the four ICC cell lines (HCCC-9810, RBE, HuCCT1, and Huh28), while among the four ICC cell lines, the HuCCT1 cell line had the highest endogenous WWP1 expression, and the other three cell lines had relatively lower endogenous WWP1 expression levels (Fig. 2A). Consequently, to investigate the biological functions of WWP1, a lentiviral shRNA vector was used to silence endogenous WWP1 expression in HuCCT1 cells, and western blotting confirmed that WWP1 was strongly downregulated (Fig. 2B). Then, the CCK-8 assay (Fig. 2C), colony formation assay (Fig. 2D), and EdU assay (Fig. 2E) were applied to evaluate the role of WWP1 on the proliferation capacity of HuCCT1 cells. Based on these results, we found that silencing endogenous WWP1 expression significantly inhibited the proliferation of HuCCT1 cells in vitro. In addition, the results of transwell assays showed that silencing WWP1 in HuCCT1 cells suppressed the migration (Fig. 2F) and invasion (Fig. 2G) of the cells in vitro. Next, we detected the effect of WWP1 on the tumorigenicity of ICC in vivo using xenograft models by subcutaneously injecting HuCCT1 cells into the flanks of nude mice (Fig. 4A–C). The data revealed that silencing WWP1 greatly inhibited tumor growth (Fig. 4A), as shown by lower tumor volumes and lower weight (Fig. 4B) in the WWP1 knockdown group compared to the control group. IHC analysis of the subcutaneous tumors showed that ki-67 was significantly decreased in WWP1-knockdown xenograft tumors compared to the negative controls (Fig. 4C). These results demonstrated that the knockdown of WWP1 in ICC cells inhibited their malignant phenotypes.

**Fig. 3:**
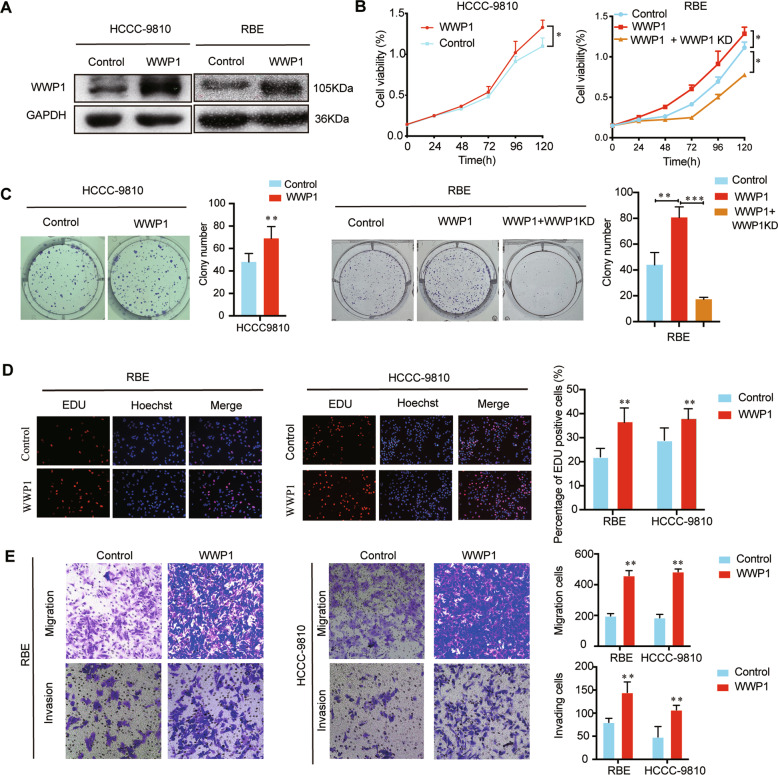
WWP1 overexpression promoted proliferation, migration, and invasion of ICC cell lines in vitro. **A** The efficiency of WWP1 overexpression was estimated by western blotting. **B** CCK-8 assay, **C** colony formation assay, **D** EdU assay were applied to estimate the proliferation of HCCC-9810 and RBE cells after WWP1 overexpression, *n* = 3. Silencing WWP1 again in RBE cells overexpressed WWP1 protein reversed the ability of proliferation (**B**, **C**). **E** Transwell assays were performed to detect the migration and invasion of HCCC-9810 and RBE cells after WWP1 overexpression. *P < 0.05, **P < 0.01.

**Fig. 4:**
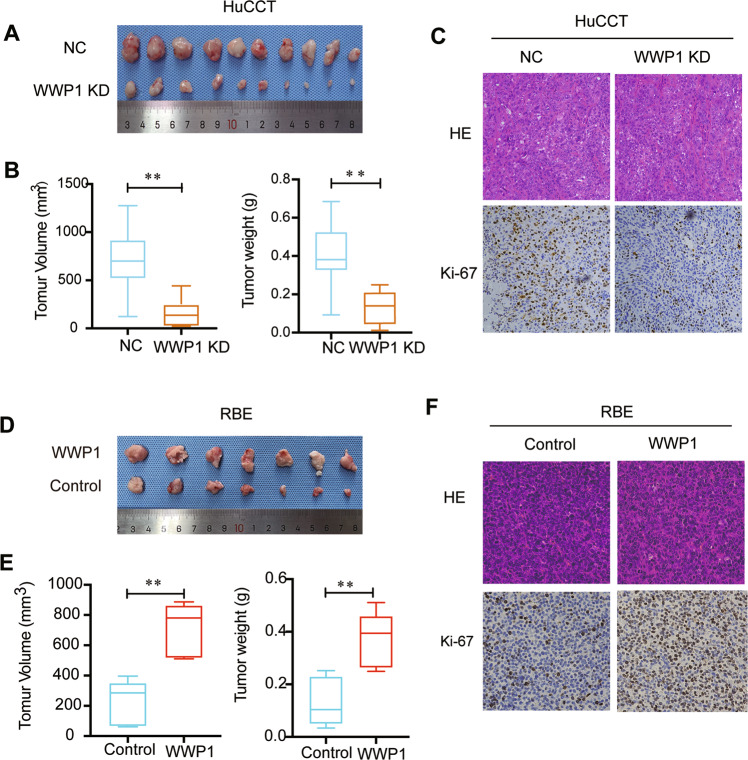
WWP1 promoted the subcutaneous tumor growth of ICC cells in vivo. **A**–**C** Subcutaneous tumor models were applied to explore the impact of WWP1 silencing on proliferation in vivo. **A** The images of tumors were generated in nude mice using WWP1 silenced HuCCT1 cells, *n* = 10. **B** The volumes and weights of subcutaneous tumors between WWP1 silencing and negative control groups. **C** HE and Ki-67 IHC staining images of subcutaneous tumors. **D**–**F** Subcutaneous tumor models were performed to detect the influence of WWP1 overexpression on proliferation in vivo. **D** The images of tumors were generated in nude mice using WWP1 overexpression RBE cells, *n* = 7. **E** The volumes and weights of subcutaneous tumors between WWP1 overexpression and vector groups. **F** HE and Ki-67 IHC staining images of subcutaneous tumors. **P < 0.01.

Moreover, to further explore the biological functions of WWP1, we transfected two of the three WWP1 low-expression cell lines, namely HCCC-9810 and RBE, with pLV5-WWP1 lentivirus, and the efficiency of overexpression was confirmed by western blotting (Fig. 3A). In the CCK-8 assay (Fig. 3B), colony formation assay (Fig. 3C), and EdU assay (Fig. 3D), WWP1 overexpression promoted the proliferation of HCCC-9810 and RBE cells in vitro. In the transwell assays, migration and invasion abilities were enhanced in HCCC-9810 and RBE cells after WWP1 overexpression (Fig. 3E). In the xenograft models, the tumor volumes and weights were higher in the WWP1 overexpression group than in the control (Fig. 4D, E). IHC showed ki-67 was increased in the WWP1 overexpression group compared to the control group (Fig. 4F). These results demonstrate that stable overexpression of WWP1 in ICC cells facilitates their proliferation, migration, and invasion.

Interestingly, overexpression of WWP1 again in HuCCT1 cells silenced endogenous WWP1 expression (Fig. 2C, D and Fig. S2A) and knocking down the WWP1 expression again in RBE cells overexpressed WWP1 protein (Fig. 3B, C and Fig. S2B) reversed the ability of proliferation in vitro, these rescue experiments further reinforced the important role of WWP1 on the proliferation of ICC cells.

### Upregulation of WWP1 in ICC was caused by MYC directly activating

**Fig. 5:**
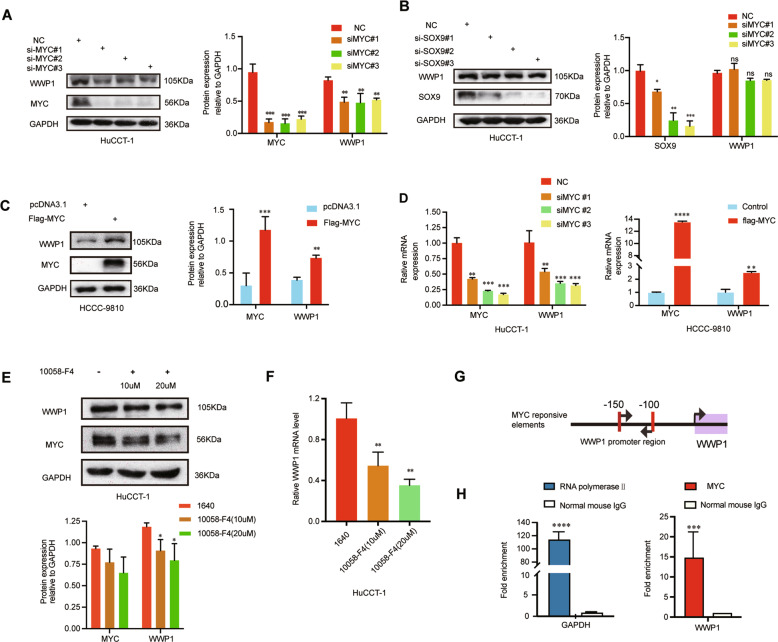
MYC transactivated WWP1 gene expression in ICC cells. **A** The protein level of WWP1 in HuCCT1 cells with MYC knockdown was evaluated by western blotting, *n* = 3 **B** The protein level of WWP1 in HuCCT1 cells with SOX-9 knockdown was determined by western blotting, *n* = 3. **C** The protein level of WWP1 in HCCC-9810 cells with MYC overexpression was evaluated by western blotting, *n* = 3. **D** The mRNA levels of WWP1 in HuCCT1 cells with MYC knockdown and HCCC-9810 cells with MYC overexpression were evaluated by qPCR, *n* = 3. **E** The protein level of WWP1 in HuCCT1 cells treated with 10058-F4, a specific c-MYC inhibitor, in the indicated concentrations for 24 h was evaluated by western blotting, *n* = 3. **F** The mRNA level of WWP1 in HuCCT1 cells treated with 10058-F4, a specific c-MYC inhibitor, in the indicated concentrations for 24 h were evaluated by qPCR, *n* = 3. **G** Schematic representation of MYC responsive elements in the promoter region of WWP1. **H** Chromatin level of MYC at the promoter region of WWP1 was detected in HuCCT1 cells. Fold enrichment of MYC relative to normal mouse IgG was evaluated by qChIP assay. Fold enrichment of RNA polymerase II at the promoter region of GAPDH served as a positive control. *P < 0.05, **P < 0.01, ***P < 0.001.

To investigate the cause of the upregulation of WWP1 in ICC, we searched for the underlying upstream mechanisms of WWP1 by retrieving information from the literature. We found that proto-oncogenic MYC and SOX9 enhanced the expression of WWP1 in other diseases [16, 21]. Next, we constructed MYC siRNA and SOX9 siRNA to interfere with MYC and SOX9 expression in HuCCT1 cells, respectively, and then used western blotting to detect the changes in WWP1expression. The results showed that the expression of WWP1 was downregulated after MYC silencing, but did not change after SOX9 silencing (Fig. 5A, B). Therefore, MYC was chosen for further investigations. HCCC-9810 cells were transfected with plasmids expressing MYC. After transfection, western blotting was used to evaluate the changes in WWP1 expression. The results showed that MYC promoted the expression of WWP1 in HCCC-9810 cells (Fig. 5C). Because MYC serves as a transcription factor, we further tested whether WWP1 was also a MYC target gene in ICC cells. Firstly, we applied the qPCR to explore whether MYC influenced the mRNA level of WWP1. The results of qPCR showed the mRNA level of WWP1 was downregulated in HuCCT1 cells after MYC silencing while that was upregulated in HCCC-9810 cells overexpressed MYC, which indicated that MYC activated the transcription of WWP1 in ICC cells (Fig. 5D). Secondly, to exclude that suppression of MYC impact on other proteins or molecular mechanisms that could influence the expression of WWP1, 10058-F4, a specific inhibitor of c-MYC, was used to detect whether there was the same result after the treatment of 10058-F4 [22, 23]. The results of 10058-F4 treatment indicated that the protein level and mRNA level of WWP1 were reduced in HuCCT1 cells incubated with 10058-F4 (Fig. 5E, F). Furthermore, we chose the HuCCT1 cell line, which has the highest WWP1 expression for chromatin immunoprecipitation (CHIP) assay to detect whether MYC directly enhances WWP1 expression. The results of the CHIP assay confirmed that endogenous MYC was significantly enriched in the promoter region of WWP1 with RNA polymerase II binding to the promoter region of GAPDH as the positive control for this CHIP assay (Fig. 5G, H). In summary, the above results suggest that the upregulation of WWP1 in ICC is caused by MYC direct activation.

### WWP1 targeted NDFIP1 for ubiquitination and degradation

**Fig. 6:**
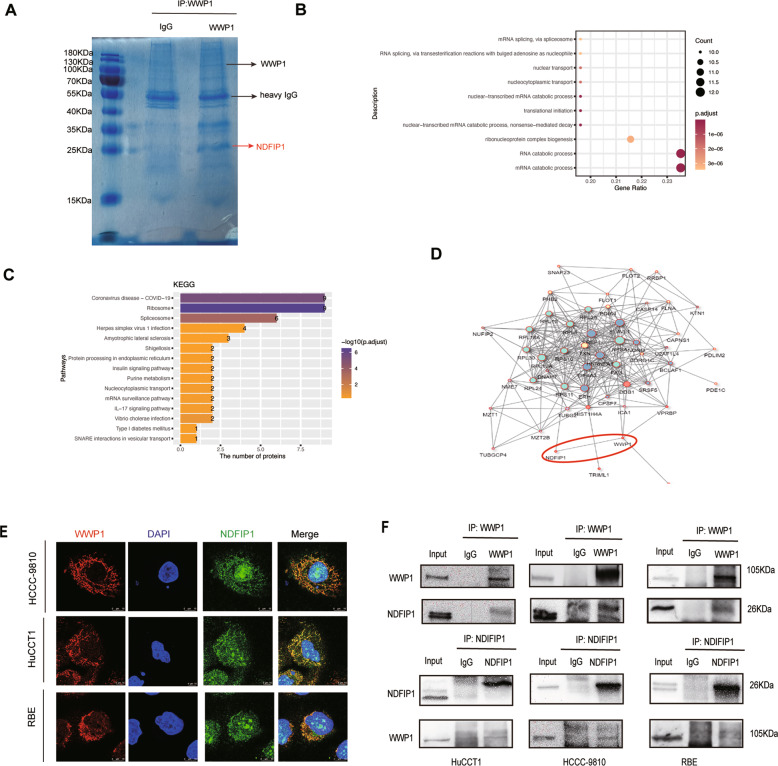
WWP1 interacted with NDFIP1. **A** Proteins that interacted with WWP1 were detected by Coomassie blue staining SDS-PAGE gel and mass spectrometry. NDFIP1 was identified as its molecular weight located on one specific band (red arrow). **B** Gene Ontology (GO) analysis of WWP1-binding proteins identified by mass spectrometry was performed. **C** WWP1 binding proteins were analyzed by KEGG pathway enrichment. **D** The protein-protein interaction network among proteins identified by mass spectrometry was constructed by PPI analysis in String Database. **E** WWP1(red) and NDFIP1 (green) colocalization in indicated cells was performed by confocal microscopy after immunofluorescence staining. **F** Interaction between WWP1 and NDFIP1 in HuCCT1, HCCC-9810, RBE cells were confirmed by co-IP assays followed by western blotting.

To explore the underlying mechanisms of WWP1 in ICC progression, we first determined the interacting proteins of WWP1 in ICC cells. WWP1 and its binding proteins were precipitated using WWP1 antibody, and the proteins that interacted with WWP1 were identified by mass spectrometry (Fig. 6A). A total of 84 proteins bound to WWP1 were identified. To illustrate the function of the WWP1-binding proteins, the gene ontology (GO) analysis and the Kyoto Encyclopedia of Genes and Genomes (KEGG) analysis of these proteins were performed in Fig. 6B, C, indicating that WWP1 might regulate fundamental cellular activities. Combined with the results of protein–protein interaction (PPI) network (Fig. 6D) with the obvious specific band at a molecular weight of 26KDa in SDS-PAGE gel, NDFIP1 was of interest because its PPxY motifs were predicted to directly bind to the WW domains of WWP1(Fig. 6A). Accordingly, we performed IF staining and co-IP assays to detect the interaction between WWP1 and NDFIP1 in ICC cell lines. From the results, WWP1 was confirmed that interact with NDFIP1 in HuCCT1, HCCC-9810, and RBE cells (Fig. 6E, F).

**Fig. 7:**
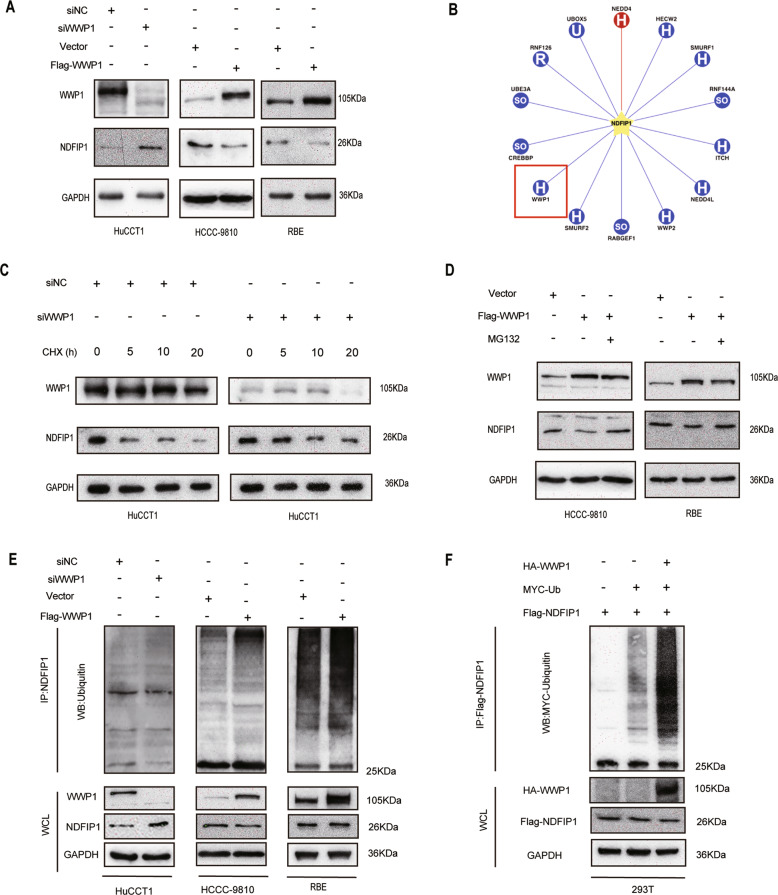
WWP1 targeted NDFIP1 for ubiquitination and degradation. **A** The protein level of NDFIP1 in HuCCT1 cells with WWP1 knockdown was evaluated by western blotting. The protein levels of NDFIP1 in HCCC-9810 and RBE cells with WWP1 overexpression were measured by western blotting. **B** Using UbiBrowser Database (http://ubibrowser.ncpsb.org/), NDFIP1 was predicted to be ubiquitinated by WWP1. **C** HuCCT1 cells infected with the lentiviral shWWP1 and control lentivirus were incubated with 100 ug/ml cycloheximide (CHX) for the indicated time. The half-life of NDFIP1 was evaluated by western blotting. **D** The protein level of NDFIP1 in HuCCT1 cells with WWP1 knockdown, HCCC-9810, and RBE cells with WWP1 overexpression treated with 20 μM MG132, a potent proteasome inhibitor, for 4 h were analyzed by western blotting. **E** Ubiquitylation levels of NDFIP1 detected by Western blotting with anti-ubiquitin antibody after WWP1 knockdown and overexpression in HuCCT1, HCCC-9810, and RBE cell lines, respectively. **F** 293T cells were transfected with the indicated plasmids, the cell lysates were incubated with Flag-tagged beads and the ubiquitylation level of Flag-NDFIP1 was detected by western blotting with an anti-MYC antibody.

Moreover, we explored whether WWP1 influenced the level of NDFIP1 via western blotting. As shown in Fig. 7A, depletion of WWP1 led to an elevation in NDFIP protein level in HuCCT1cells, and upregulation of WWP1 led to a decrease in NDFIP1 protein levels in HCCC-9810 and RBE cells, indicating that WWP1 is involved in controlling NDFIP1 stability. Given that WWP1 is an E3 ubiquitin-protein ligase, we suspected that NDFIP1 might be a ubiquitination substrate of WWP1. Synchronously, we screened the UbiBrowser database and found that NDFIP1 was predicted to be ubiquitinated by WWP1 (Fig. 7B). Therefore, we further explored whether WWP1 regulated NDFIP1 levels through ubiquitination. Firstly, we investigated whether WWP1 could affect the half-life of NDFIP1. Cells were treated with 100ug/ml CHX for inhibition of protein biosynthesis and harvested at the indicated time points. We found that silencing of WWP1 in HuCCT1 cells extended the half-life of NDFIP1, indicating that WWP1 influenced the degradation of NDFIP1 in ICC cells (Fig. 7C). Secondly, we found that the inhibition of WWP1 on NDFIP1 could be rescued by MG132, a proteasome inhibitor (Fig. 7D). This result further suggested that ubiquitination might be the main process of this regulation. Accordingly, the ubiquitination assays were performed and the results revealed that knockdown of WWP1 reduced the ubiquitination of NDFIP1 in HuCCT1 and vice versa in HCCC-9810 and RBE (Fig. 7E). Synchronously, the identical result was reinforced in 293T cells (Fig. 7F). Taken together, our data suggest that WWP1 promotes the ubiquitination and degradation of NDFIP1.

### WWP1 performed its tumorigenic function via downregulation of NDFIP1 in ICC cells

**Fig. 8:**
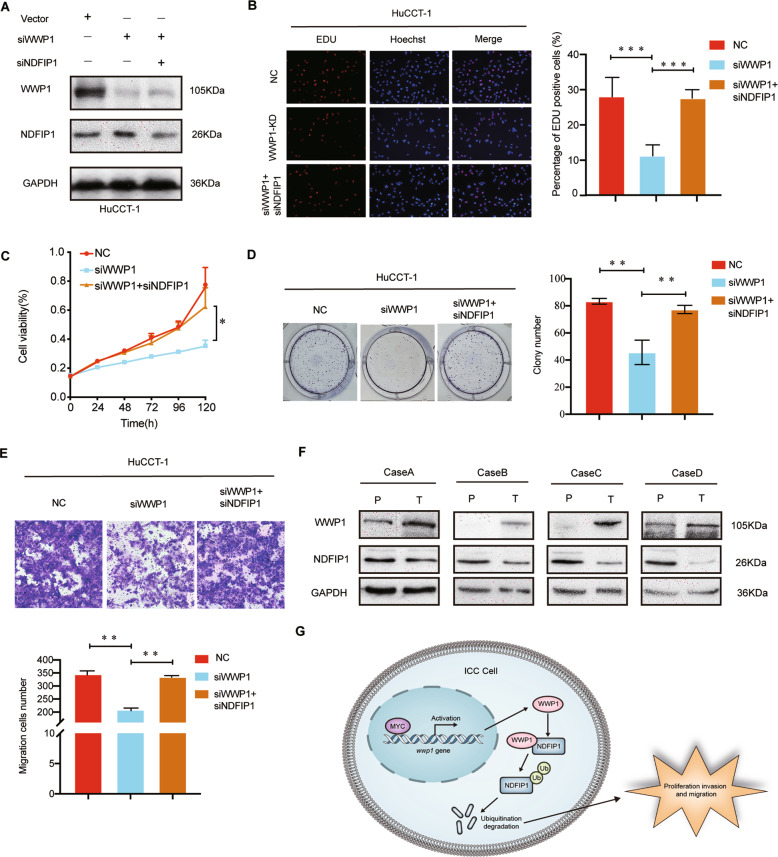
WWP1 promoted ICC proliferation and migration via negative regulation of NDFIP1. **A** HuCCT1 cells were transfected with WWP1 siRNA and NDFIP1 siRNA, then western blotting was used to detect the efficiency of transfection. **B**–**D** HuCCT1 cells were transfected with WWP1 siRNA and NDFIP1 siRNA, **B** EdU assay, **C** CCK-8 assay, and **D** colony formation assay were applied to estimate the proliferation of HuCCT1, *n* = 3. **E** HuCCT1 cells were transfected with indicated siRNA. Transwell assays were performed to detect the migration of HuCCT1, *n* = 3. **F** Images of WWP1 protein levels and NDFIP1protein levels in 4 identical ICC tumors and paired adjacent non-tumor tissues by western blotting assays. **G** A proposed model of how WWP1 regulates the ICC progression: MYC upregulates WWP1 in ICC cells, thereby targeting NDFIP1 for ubiquitination and degradation to promote ICC cell proliferation, migration, and invasion. **P < 0.01, ***P < 0.001.

To further confirm whether NDFIP1 was necessary for the tumorigenic function of WWP1 in ICC cells, HuCCT1 cells were transfected with WWP1 siRNA, NDFIP1 siRNA, or both (Fig. 8A). The results of EdU assay (Fig. 8B), CCK-8 (Fig. 8C), and colony formation assay (Fig. 8D) conformed that downregulation of NDFIP1 reversed the defects in the proliferation of HuCCT1 cells with WWP1 knockdown. Consistently, NDFIP1 silencing rescued the weakened metastasis of HuCCT1 after WWP1 downregulation (Fig. 8E). Additionally, we detected the protein levels of WWP1 and NDFIP1 in the four patient tissues. Western blotting showed that the levels of WWP1 were elevated in tumor tissues with low expression levels of NDFIP1 (Fig. 8F), indicating a negative correlation between WWP1 expression and NDFIP1 in clinical cases.

## Discussion

Recently, the incidence of intrahepatic cholangiocarcinoma (ICC) has dramatically increased, however, the treatments for ICC are limited and unsatisfactory. Therefore, there is an urgent need to elucidate the molecular mechanisms that drive malignant progression and identify novel biomarkers for this disease. The present study demonstrated that WWP1 was upregulated by MYC in ICC, and the expression of WWP1 was associated with poor clinical prognosis. Overexpression of WWP1 induced proliferation, migration, and invasion of ICC cell lines, and suppression of WWP1 induced opposite events. Mechanistically, we identified the interaction between NDFIP1 and WWP1 in ICC. In addition, we observed that WWP1 targeted NDFIP1 for ubiquitination and degradation, which might be a novel mechanism for ICC progression. Generally, our study clearly showed the critical role of WWP1 in the proliferation and metastasis of ICC cells, suggesting that WWP1 plays an oncoprotein function that facilitates ICC progression.

WWP1 is a member of the NEDD4-like family which belongs to the HECT-type E3 ubiquitin ligases (HECT E3s). Recently, many studies have demonstrated that aberrant expression of multiple HECT E3s is closely associated with the progression of various human cancers [6, 24]. For instance, NEDD4, a classical member of the HECT family, has been reported to play an oncogenic role in the diversity of carcinomas, such as breast, lung, bladder, and gastric cancers. Simultaneously, WWP1 and WWP2 have also been confirmed to serve as tumorigenic capacity by controlling several core cellular pathways. More importantly, the HECT E3s have been found to be crucial targets for therapeutic intervention. Aronchik and his colleagues [25] reported that indole-3-carbinol (I3C), a compound derived from cruciferous vegetables, could inhibit the functions of NEDD4-1 and WWP1 through targeting the HECT domain, thus abrogating the proliferation and metastasis of melanoma or prostatic carcinoma. Accordingly, our findings highlight the promising prospects of clinical applications in which WWP1 may become a new prognostic biomarker and therapeutic target for ICC.

To identify the underlying downstream mechanisms of WWP1 in ICC, we identified NDFIP1 as a novel interacting protein of WWP1 by label-free mass spectrometry. Furthermore, we also revealed that WWP1 regulated the ubiquitination level of NDFIP1, thus resulting in its degradation, which suggests that NDFIP1 is a ubiquitination substrate of WWP1. NDFIP1 is an adaptor of Nedd4-family ubiquitin ligases. Kieran F et al.demonstrated that NDFIP1 could bind to many members of the Nedd4 family, such as NEDD4, smurf1, and WWP2, and was ubiquitinated by them, which was in line with our findings [26]. Recently, several studies have reported that NDFIP1 acts as a critical tumor suppressor. In pancreatic ductal adenocarcinoma and HCC, NDFIP1 downregulation by miRNAs promotes tumor progression or epithelial-mesenchymal transition [20, 27]. However, there is no study has reported on the function of NDFIP1 in ICC. The present data suggest that downregulation of NDFIP1 by WWP1-induced ubiquitination promoted proliferation and invasion of ICC cells. Accordingly, our study provides more evidence that NDFIP1 functions in tumor inhibition. In addition, our data also revealed that WWP1 and NDFIP1 are negatively correlated in ICC tissues, further indicating the oncogenic capacity of the WWP1/NDFIP1 axis in ICC progression.

Apart from the downstream pathways of WWP1 in ICC, we also detected the upstream causes upregulation of WWP1 in ICC. Previous reports revealed that multiple miRNAs and transcription factors regulate the expression of WWP1, such as miR-584-5p, miR-30a-5p, miR-16-5p, KLF2/5, MYC, SOX9, and so on [16, 21, 28–31]. Among these, MYC was of our interest, because MYC is proved to be a critical oncogene that drives many types of cancer tumorigenesis. More importantly, aberrancies of the MYC-WWP1-PTEN signaling axis were determined to be a core axis in the progression of cancers [16]. Accordingly, we confirmed that MYC was enriched in the promoter region of WWP1 in ICC cells, providing novel insights into the mechanisms of ICC proliferation and tumorigenesis.

In summary, this study demonstrated that WWP1 was upregulated in ICC tumor tissues compared to paired non-tumor tissues, and high WWP1 expression was associated with a poor prognosis. For mechanisms, we clarified that the upregulation was caused by MYC combined in the promoter of WWP1 to enhance its expression. Simultaneously, we revealed a tumorigenic role of WWP1 in ICC that could promote proliferation, migration, and invasion of ICC cells by regulating the ubiquitination of NDFIP1, providing a potential therapeutic strategy for patients with ICC.

## Materials and methods

### Patients and specimens

Clinical specimens from 87 patients with ICC were obtained from Peking University People’s Hospital (Beijing, China). All patients underwent hepatic resection between January 2015 and September 2019. Patients who were previously diagnosed with other carcinomas and who received neoadjuvant treatments before surgery were excluded. Histopathological diagnoses were made according to the WHO criteria. All patients were examined routinely after surgery and were followed up until September 2021. Clinicopathological data of all patients were retrospectively collected from the hospital records. In addition, among the ICC patients who underwent surgery between October 2019 and September 2021 at our hospital, 10 case and 20 cases were randomly enrolled to perform western blotting and IHC respectively to evaluate the expression level of WWP1. Written informed consent was obtained from the donors in this study for use of their tissues prior to the acquisition of the specimens. Ethical approval was granted by the Research Ethics Committee of Peking University People’s Hospital.

### Cell culture and reagents

Normal human intrahepatic biliary epithelial cell (HIBEC) (purchased from Zhejiang Meisen Cell Technology Co., Ltd, Hangzhou, China). Human ICC cell lines included RBE and HCCC-9810 (purchased from China Infrastructure of Cell Line Resource, Beijing, China), Huh28 (purchased from Zhejiang Meisen Cell Technology Co., Ltd, Hangzhou, China), and human ICC cell line HuCCT-1 (obtained from the Chinese Academy of Science, Shanghai, China), 293T cell (purchased from Bo’ao Kaimei Technology, Beijing, China). All ICC cell lines and 293T cells were authenticated using STR in qualified institutions. HIBEC was cultured in a primary cell culture medium (purchased from Zhejiang Meisen Cell Technology Co., Ltd, Hangzhou, China). All ICC cells and 293T cells were cultured in RPMI 1640 (Biological Industries, Israel) supplemented with 10% (FBS bovine serum FBS (Gibco, USA) and 1% penicillin–streptomycin (Biological Industries, Israel) at 37 °C under 5% CO_2_.

Anti-WWP1 antibody (SAB2102717) for immunohistochemistry (IHC) analysis was purchased from Millipore Sigma (Billerica, MA, USA). Anti-WWP1 antibody (ab43791) for immunoblotting and IF analysis and Anti-WWP1 antibody (ab104440) for immunoprecipitation were purchased from Abcam (Cambridge, UK). Anti-MYC tag antibody (ab32072), Anti-SOX9 antibody (ab185966) were purchased from Abcam (Cambridge, UK). Normal rabbit IgG (#2729S) was purchased from Cell Signaling Technology (Danvers, MA, USA). The NDFIP1 antibody (15602-1-AP) was obtained from Proteintech (Wuhan, China). Anti-Multi ubiquitin antibody (D058-3) was purchased from MBL Life Science (Tokyo, Japan). 10058-F4(HY-12702) was purchased from MedChemExpress (New Jersey, USA)

### Animals experiments

The BALB/c nu/nu mice (male, 4 weeks, 15–20 g) were purchased from Vital River Laboratories (Beijing, China). All mice were housed in the experimental animal center of Peking University People’s Hospital. One experimental group contained 10 mice. For xenograft tumor models, nude mice were injected subcutaneously in the flank with 6 × 10^6^ indicated cells suspended in 100 μL PBS. Then these mice were observed every three days. Until 6 weeks later, the mice were sacrificed. The xenograft tumors were excised from subcutaneous tissue and the tumor weight and volumes were measured. All animal experiment protocols and procedures were approved by the Ethical Review Committee of Peking University People’s Hospital.

### RNA interference

All siRNAs were synthesized by XIEBHC Biotechnology (Beijing, China) and the sequences of the siRNAs are shown in Table 2. Lipofectamine RNAiMAX reagent (13778030, Invitrogen) was used for transfection in 6-well plates. 48 h after transfection, the cells were collected and analyzed by western blotting.

**Table 2:** Sequences of small interference RNA.

Name	Sequences
MYC siRNA #1	Sense: 5’-CGACGAGACCUUCAUCAAAdTdT-3’
	Antisense: 5’-UUUGAUGAAGGUCUCGUCGdTdT-3’
MYC siRNA #2	Sense: 5’-CGAUGUUGUUUCUGUGGAAdTdT-3’
	Antisense: 5’-UUCCACAGAAACAACAUCGdTdT-3’
MYC siRNA #3	Sense: 5’-GGAACUAUGACCUCGACUAdTdT-3’
	Antisense: 5’-UAGUCGAGGUCAUAGUUCCdTdT-3’
SOX9 siRNA #1	Sense: 5’-GGCUGCGCGUGCAGCACAAdTdT-3’
	Antisense: 5’-UUGUGCUGCACGCGCAGCCdTdT-3’
SOX9 siRNA #2	Sense: 5’-GGAGGAAGUCGGUGAAGAAdTdT-3’
	Antisense: 5’-UUCUUCACCGACUUCCUCCdTdT-3’
SOX9siRNA #3	Sense: 5’-GCAGCGACGUCAUCUCCAAdTdT-3’
	Antisense: 5’-UUGGAGAUGACGUCGCUGCdTdT-3’
NDFIP1 siRNA	Sense: 5’-CCACCUUACAGCAGCAUUUdTdT-3
	Antisense: 5’-AAAUGCUGCUGUAAGGUGGdTdT-3’
WWP1 siRNA	Sense: 5’-GCAGAGAAAUACUGUUUAUUU-3’
	Antisense: 5’-AAAUAAACAGUAUUUCUCUGC-3’
Negative control siRNA	Sense: 5’-UUCUCCGAACGUGUCACGUdTdT-3
	Antisense: 5’-ACGUGACACGUUCGGAGAAdTdT-3’

### Co-immunoprecipitation (Co-IP)

Cells were lysed in Pierce IP Lysis Buffer (87788, Thermo) with a protease inhibitor mixture (P6730, Solarbio) and phosphatase inhibitor (P1260, Solarbio). Protein A/G Magnetic Beads (HY-K0202, MCE) were added to the mixture for 180 min at 4 °C and then immunoprecipitated with specific antibodies overnight at 4 °C. Then, the protein-antibody complexes were incubated with Protein A/G Magnetic Beads for another 2–4 h, the protein–magnetic beads complexes were harvested by The Magna GrIPTM Rack (#20-400, Millipore). Finally, the magnetic beads were eluted by boiling in 1× SDS-PAGE loading buffer before western blotting.

### Ubiquitination assay

The ubiquitination assay was conducted based on the previous reports [32–35]. Briefly, to detect NDFIP1 ubiquitination in ICC cells, cells were treated with 20 μM MG132 (HY-13259, MCE) for 4 h, then the cells were lysed in SDS lysis buffer (0.1% SDS, 150 mM NaCl, 10 mM Tris-HCl (pH = 7.5), 0.5% NP-40) with protease inhibitor mixture, a phosphatase inhibitor, and 10 μM *N*-ethylmaleimide (NEM, HY-D0843, MCE), The lysates were incubated with anti-NDFIP1 antibody overnight with rotation at 4 °C. The following steps were the same as the Co-IP assay described above.

To detect the NDFIP1 ubiquitination in 293T cells, cells were transfected with Flag-NDFIP1, MYC–Ub, or HA-WWP1 for 48 h, and the same methods described above was performed to lyse 293T cells treated with MG132. Then the lysates were incubated with Flag Magnetic beads (FNM-25, BEIJING LABLEAD BIOTECH CO. LTD.) for 2–4 h with rotation at 4 °C, the following steps were the same as the Co-IP assay described above.

### Chromatin immunoprecipitation

The ChIP Kit-One Step (ab117138, Abcam) was used for ChIP analysis, and all the steps were conducted according to the manufacturer’s protocol. Briefly, the freshly trypsinized cells were cross-linked with 1% formaldehyde/culture medium solution on a rocking platform for 10 min at room temperature and quenched with glycine. Chromatin extraction was performed using a Chromatin Extraction Kit (ab117152, Abcam). Then, the chromatin fractions were incubated with anti-MYC antibody (ab56, Abcam), normal mouse IgG, or RNA polymerase II antibody (both provided by the ChIP Kit-One Step) in provided wells on an orbital shaker at room temperature for 90–120 min. After the release and purification of the DNA, real-time PCR was used for further analysis.

### Immunofluorescence

The slices that contain paraffin-embedded tissues were dewaxed in dimethylbenzene, hydrated by ethylalcohols, repaired with EDTA (pH = 8.0). Then, the slices were blocked and permeabilized with blocking buffer (PBS containing 10% normal donkey serum,1% BSA, 0.1% Tween, 0.1% TritonX-100) for 1 h at room temperature. Next, the slices were incubated with specific primary antibodies overnight at 4 °C. After incubation with the secondary antibodies (Alexa Fluor 555 donkey anti-mouse IgG(H + L), A-31570, Invitrogen; Alexa Fluor 488 donkey anti-rabbit IgG(H + L), A-21206, Invitrogen) for 1 h and DAPI (C0065, Solarbio) for 10 min, the slices were imaged by confocal microscopy.

### Statistical analyses

Statistical analyses were performed using SPSS 27.0 software (IBM, Armonk, NY, USA) and GraphPad Prism 9. Continuous variables are expressed as mean ± standard deviation (SD), and categorical variables are presented as numbers and percentages. Student’s *t* test was applied to compare the differences between two groups that were made up of continuous variables. Chi-square tests were performed to analyze the clinicopathological characteristics of the enrolled patients. Survival curves were analyzed using the Kaplan–Meier method and log-rank test. Statistical significance is indicated as *P < 0.05, **P < 0.01, ***P < 0.001, n.s. *P* > 0.05.

## Supplementary information

Supplementary Information

Author Contributions Statement

WWP1 binding Proteins detected by mass spectrometry

## Data Availability

The data generated or analyzed in this study are all included in this published article. The raw data that support the findings of this study are available from the corresponding author upon reasonable request.
