# Supplementary material for: WWP1 upregulation predicts poor prognosis and promotes tumor progression by regulating ubiquitination of NDFIP1 in intrahepatic cholangiocarcinoma
Source: Cell Death Discov. 2022 Mar 9;8:107. doi: 10.1038/s41420-022-00882-0 (PMC8906119; doi:10.1038/s41420-022-00882-0)
Supplement: Supplementary file 1 — Supplementary Information [file 41420_2022_882_MOESM1_ESM.pdf]

Supplementary Information

Supplementary figures:

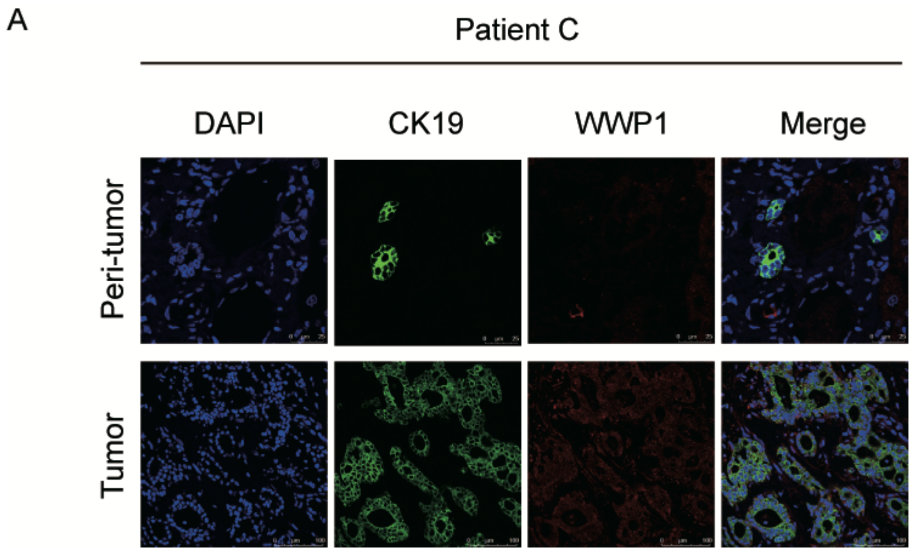

Fig. S1 WWP1 was upregulated in ICC and its high-expression was relationship with poor prognosis.

A Images of WWP1 expression levels in CK19 positive tumor cells and CK19 positive normal epithelial cells of adjacent non-tumor liver tissues by immunofluorescence staining (blue: DAPI; green: CK19; red: WWP1).

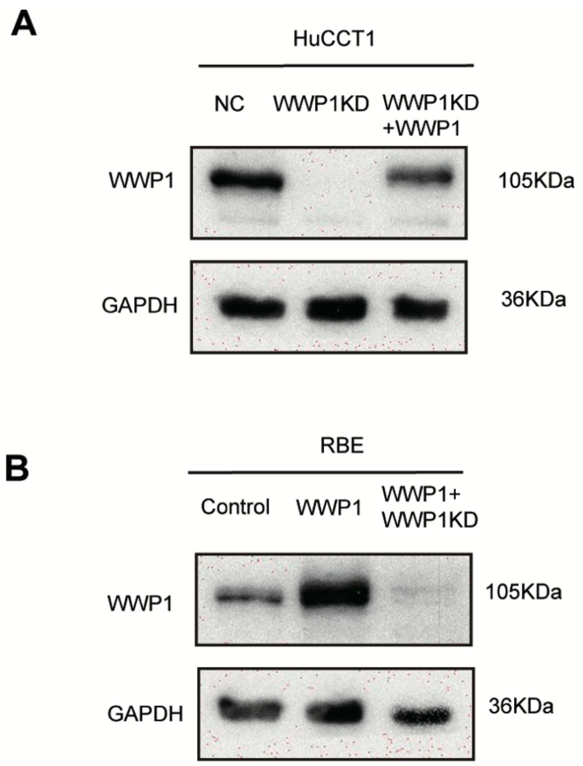

Fig. S2. Evaluation of the efficiencies of transfection in WWP1 rescued experiments.

A HuCCT1 cells with WWP1 knocked down were transfected with the lentivirus stably overexpressing WWP1 again, then western blotting was used to detect the efficiency of transfection. **B** RBE cells with WWP1 overexpression were transfected with the lentiviral shWWP1 again, then western blotting was used to detect the efficiency of transfection.

Uncropped western blots

Fig.1

Fig.1. A. Case1 WWP1

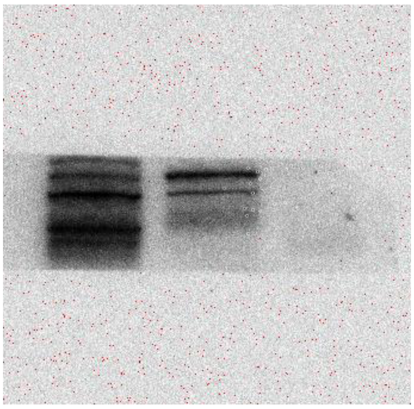

Fig.1. A. Case1 GAPDH

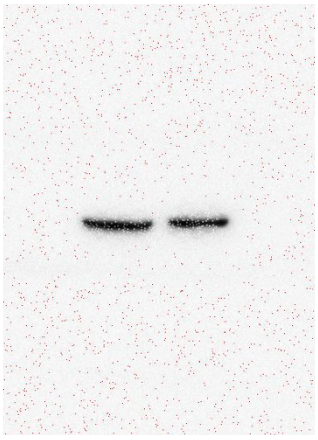

Fig.1. A. Case2 WWP1

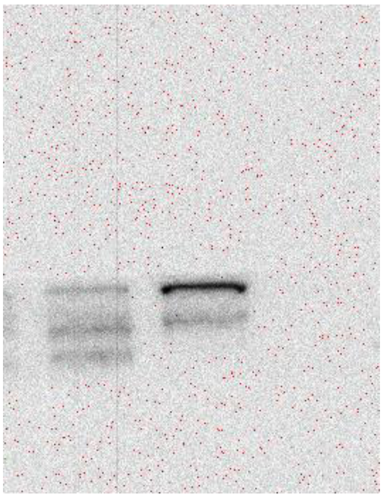

Fig.1. A. Case2 GAPDH

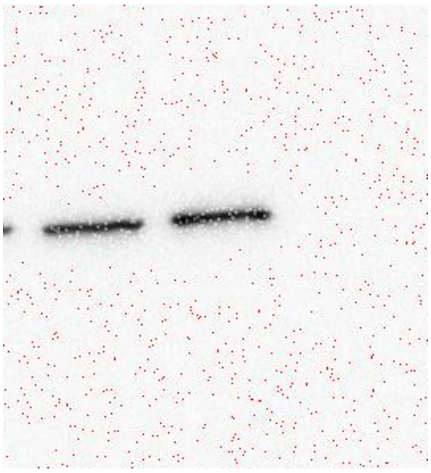

Fig.1. A. Case3 WWP1

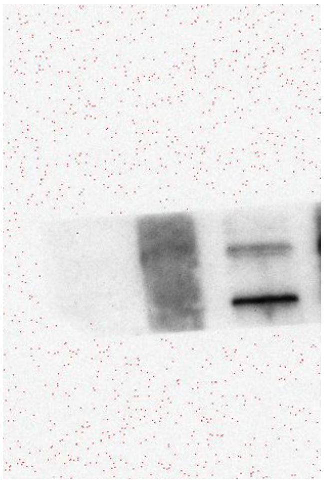

Fig.1. A. Case3 GAPDH

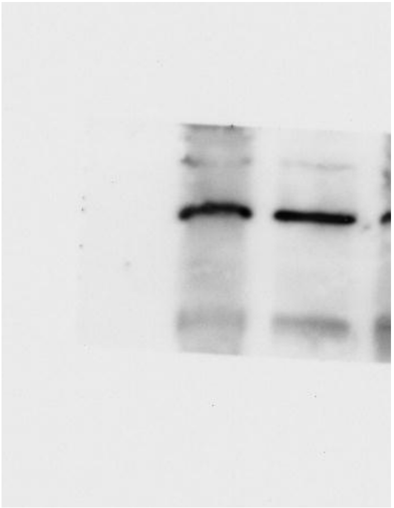

Fig.1. A. Case4 WWP1

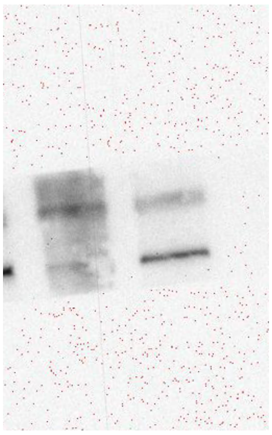

Fig.1. A. Case4 GAPDH

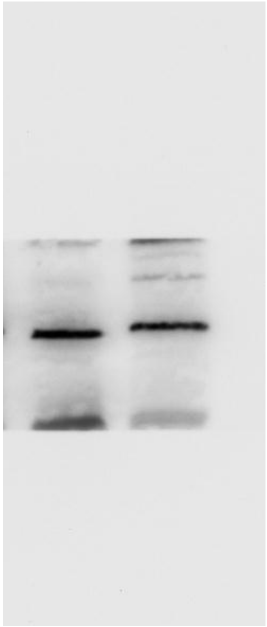

Fig.1. A. Case5 WWP1

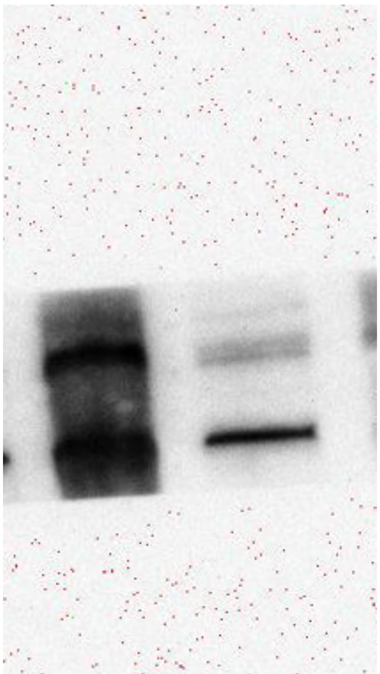

Fig.1. A. Case5 GAPDH

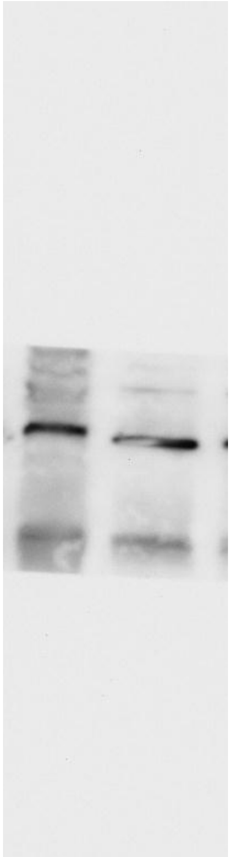

Fig.1. A. Case6 WWP1

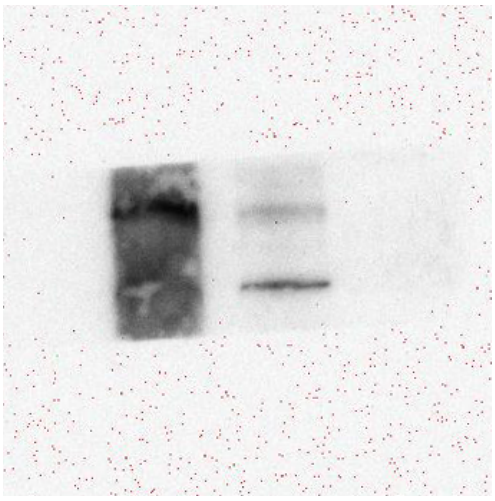

Fig.1. A. Case6 GAPDH

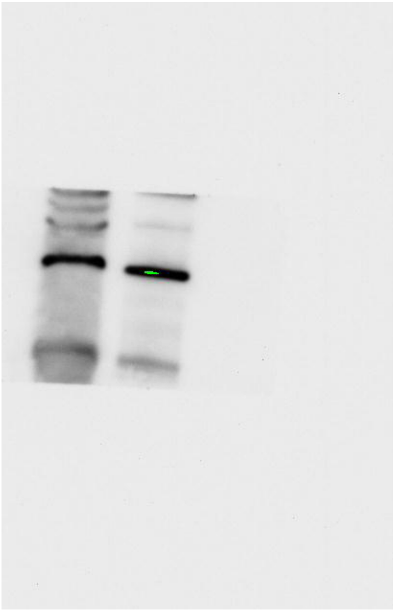

Fig.1. A. Case7 WWP1

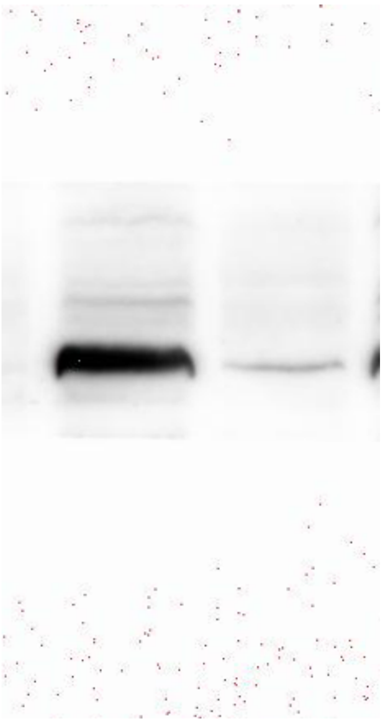

Fig.1. A. Case7 GAPDH

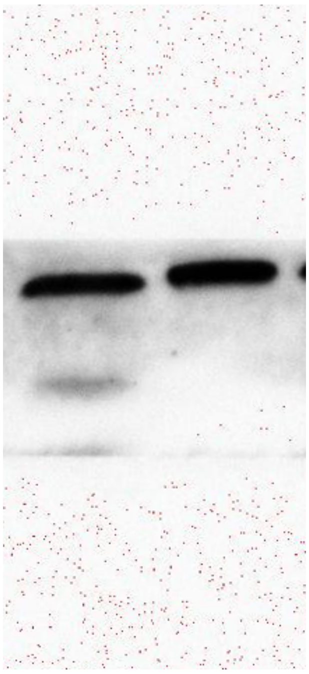

Fig.1. A. Case8 WWP1

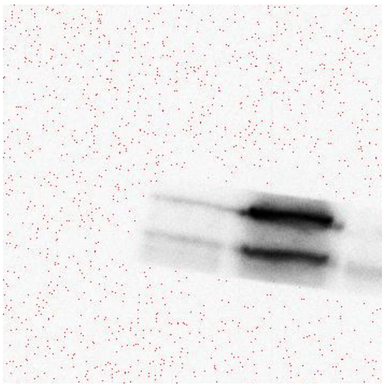

Fig.1. A. Case8 GAPDH

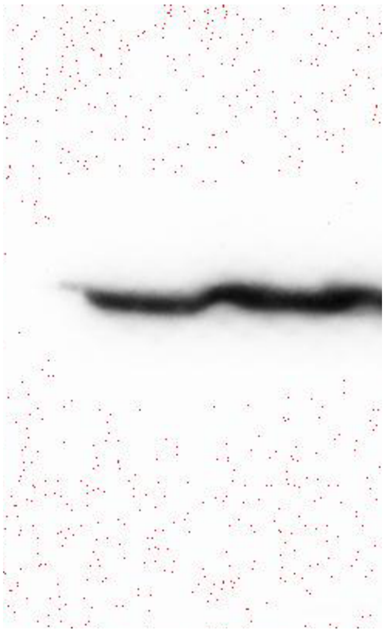

Fig.1. A. Case9 WWP1

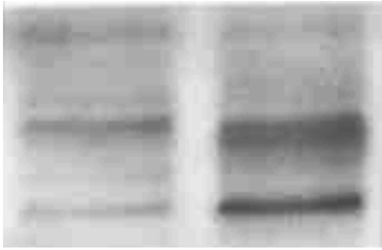

Fig.1. A. Case9 GAPDH

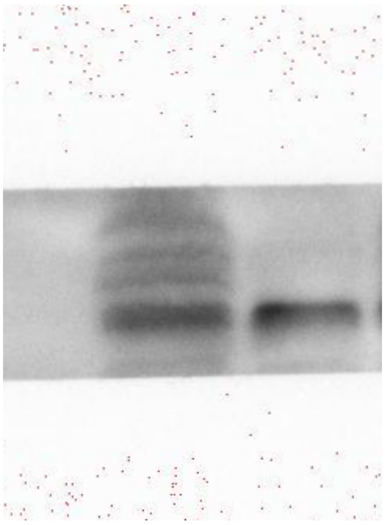

Fig.1. A. Case10 WWP1

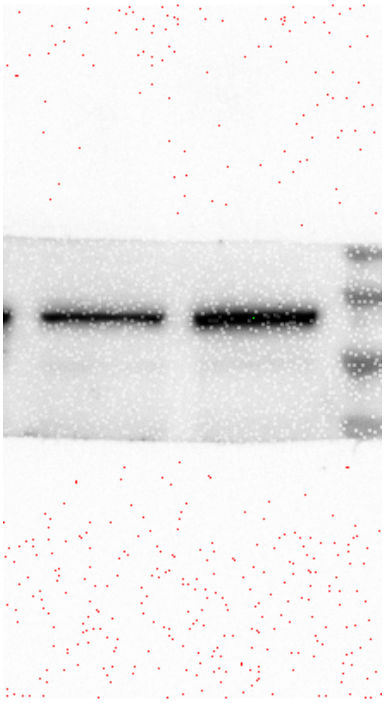

Fig.1. A. Case10 GAPDH

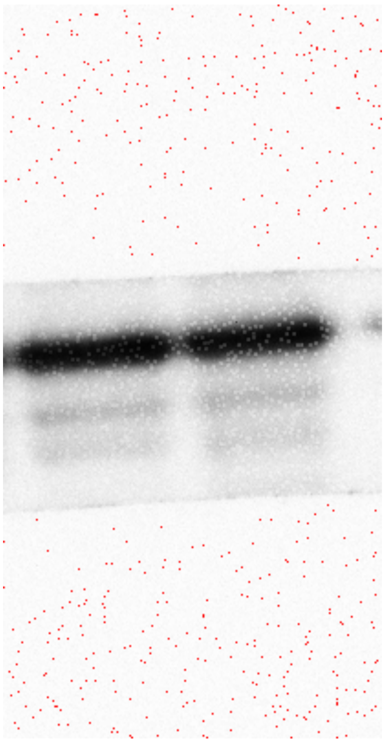

Fig.2-3

Fig.2. A. WWP1

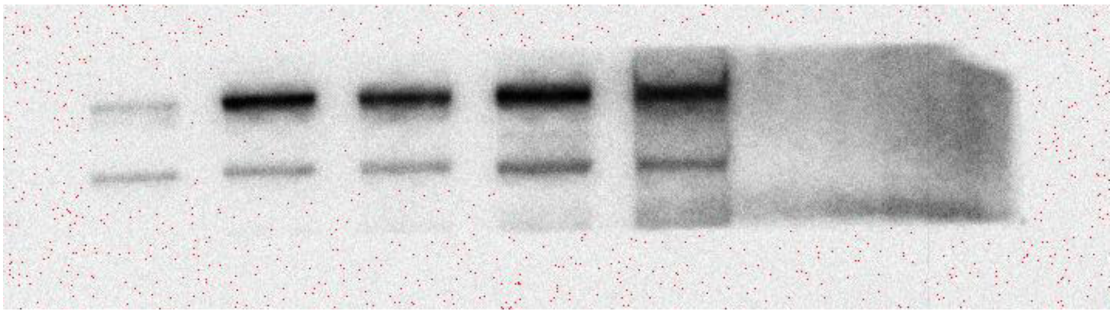

Fig.2. A. GAPDH

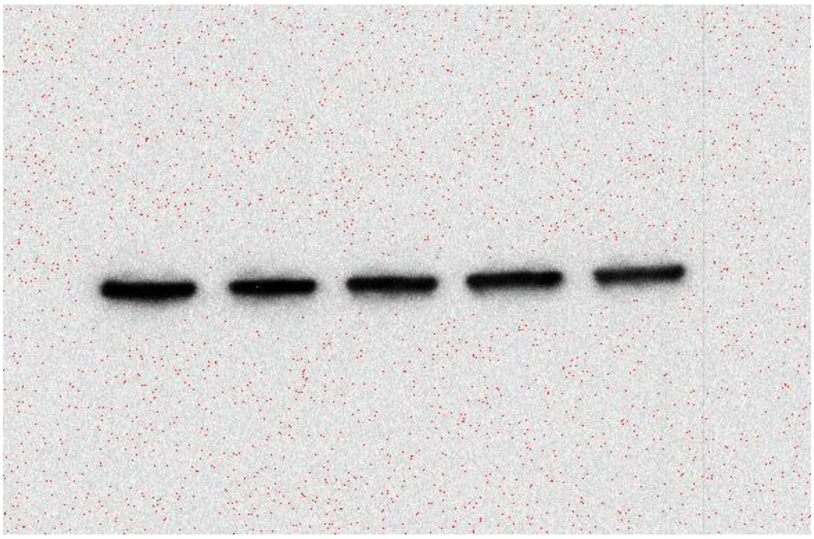

Fig.2.B. WWP1

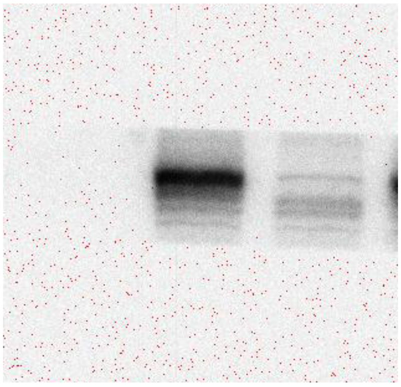

Fig.2.B. GAPDH

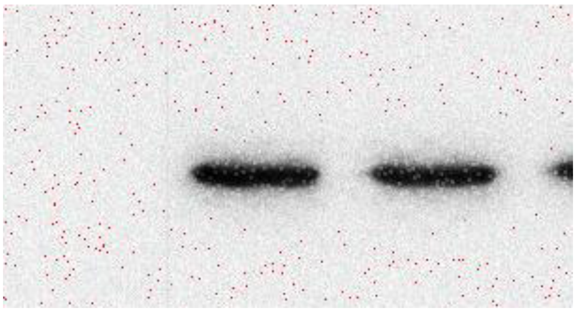

Fig.3. A. HCCC-9810 WWP1

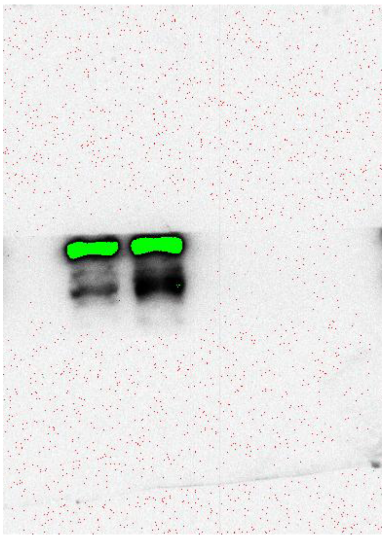

Fig.3. A. HCCC-9810 GAPDH

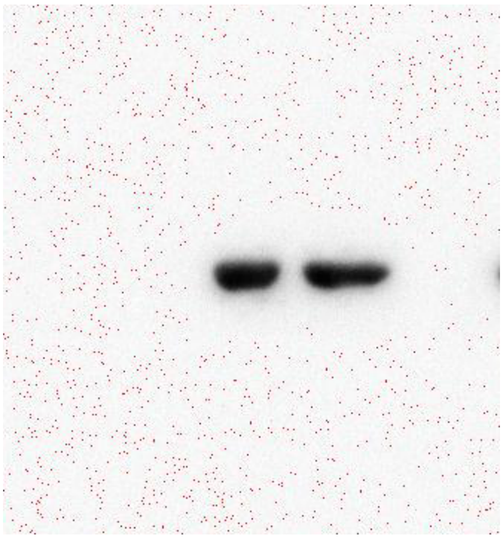

Fig.3. A. RBE WWP1

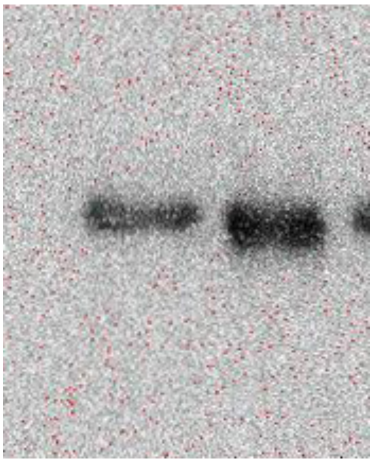

Fig.3. A. RBE GAPDH

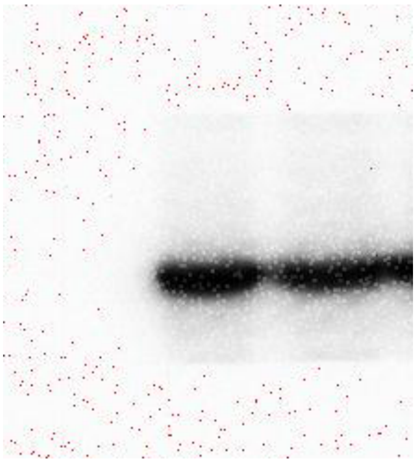

Fig.5

Fig.5. A. WWP1

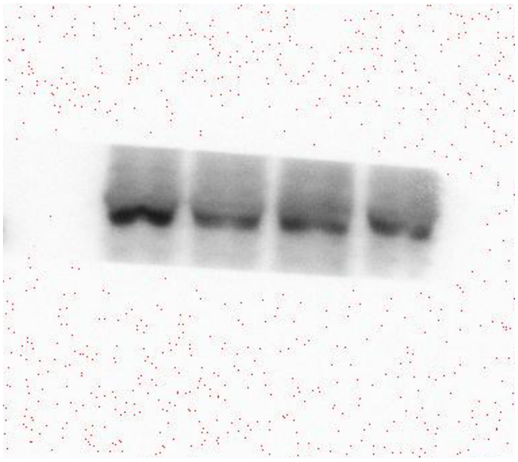

Fig.5. A. MYC

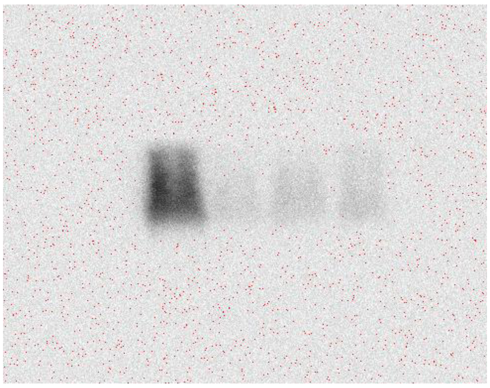

Fig.5. A. GAPDH

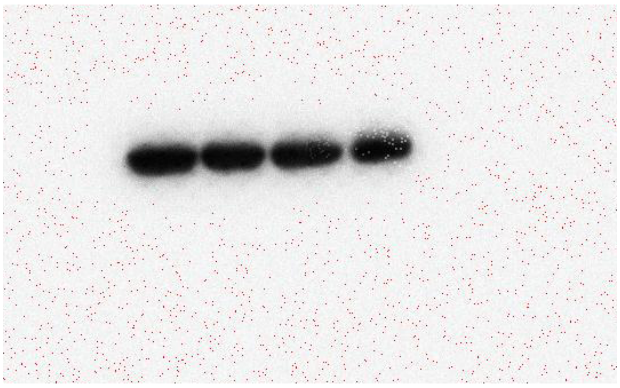

Fig.5. B WWP1

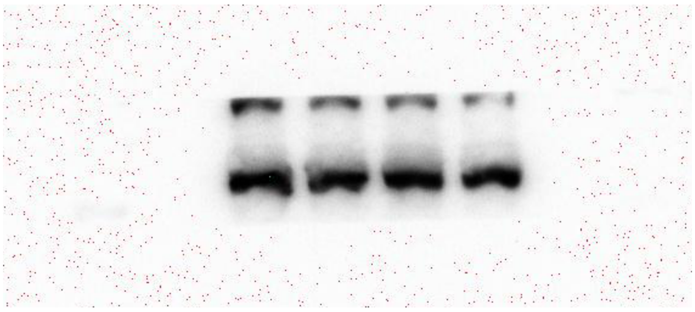

Fig.5. B. SOX9

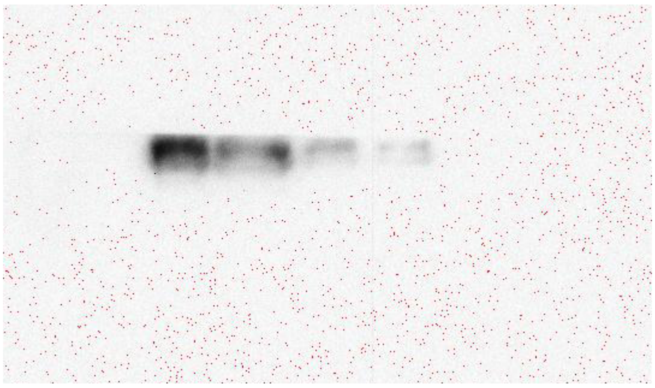

Fig.5. B GAPDH

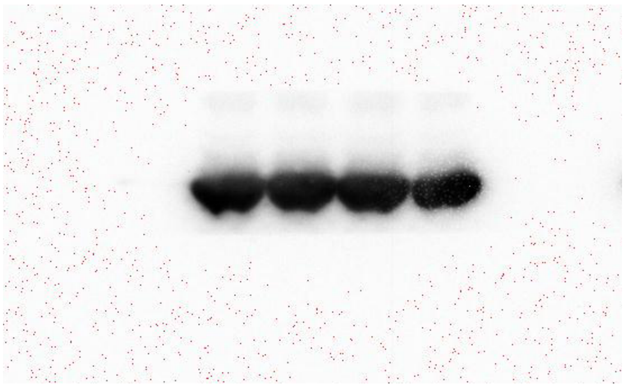

Fig.5. C. WWP1

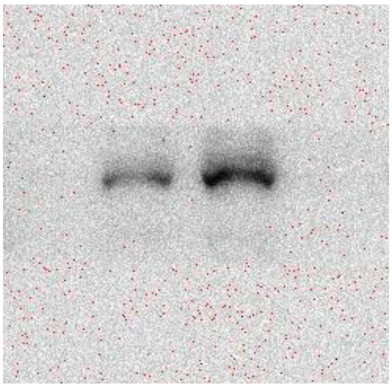

Fig.5. C. MYC

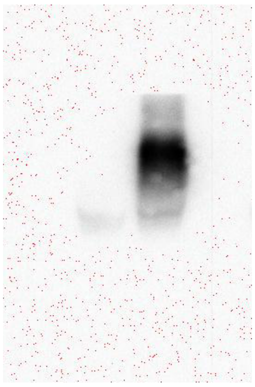

Fig.5. C GAPDH

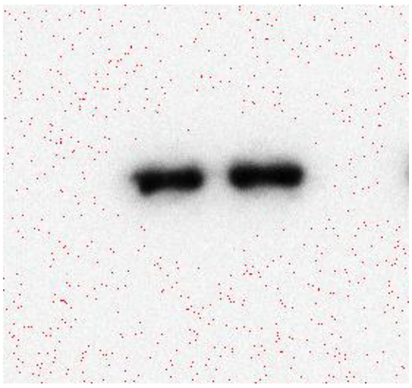

Fig.5. E. WWP1

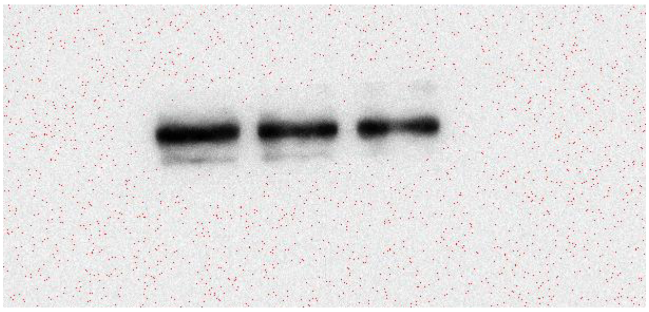

Fig.5. E. MYC

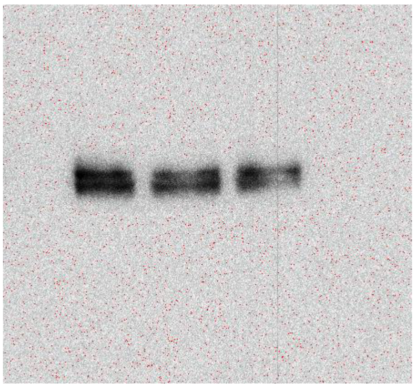

Fig.5. E. GAPDH

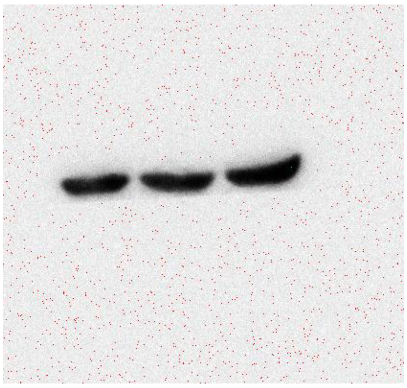

Fig.6

Fig.6. F. HuCCT1 IP:WWP1 WWP1

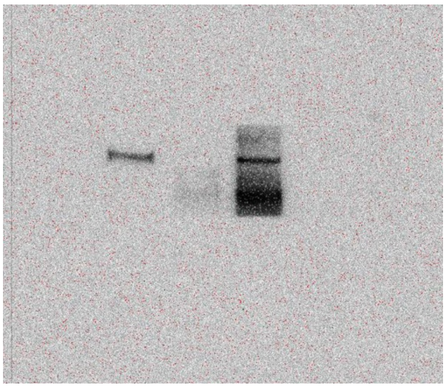

Fig.6. F. HuCCT1 IP:WWP1 NDFIP1

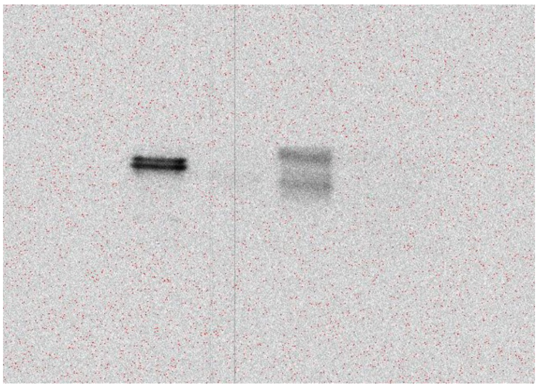

Fig.6. F. HuCCT1 IP: NDFIP1 NDFIP1

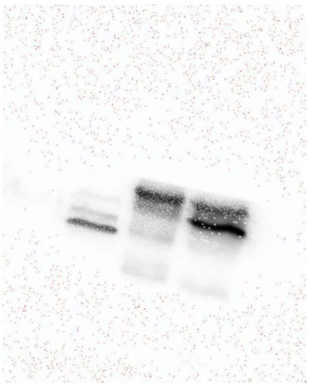

Fig.6. F. HuCCT1 IP: NDFIP1 WWP1

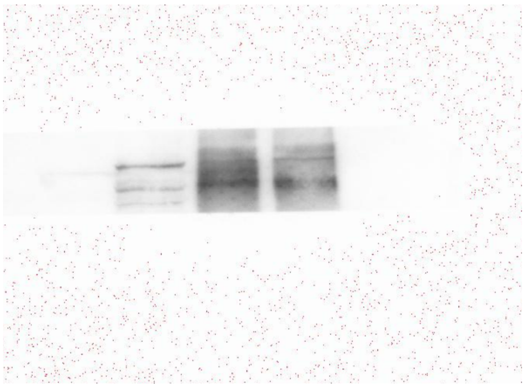

Fig.6. F. HCCC-9810 IP: WWP1 WWP1

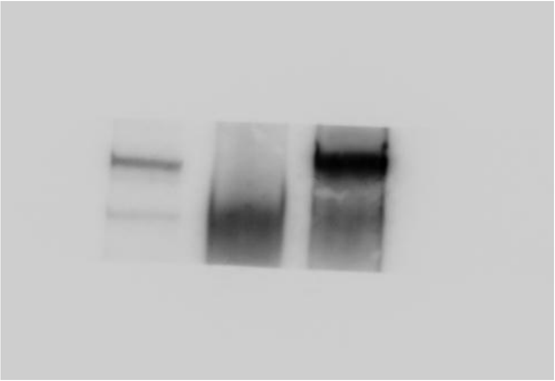

Fig.6. F. HCCC-9810 IP: WWP1 NDFIP1

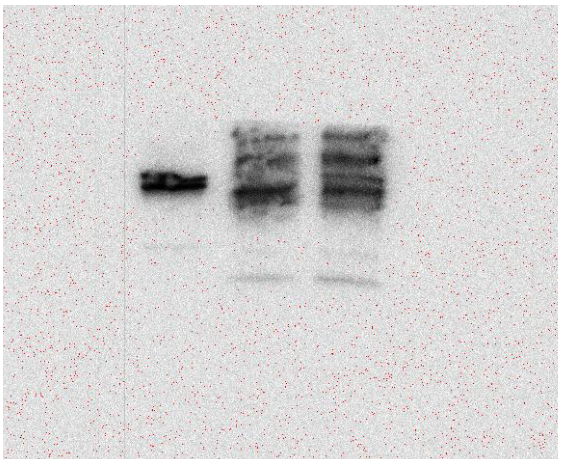

Fig.6. F. HCCC-9810 IP: NDFIP1 NDFIP1

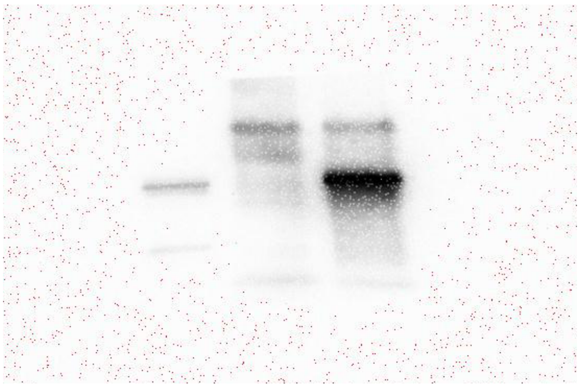

Fig.6. F. HCCC-9810 IP: NDFIP1 WWP1

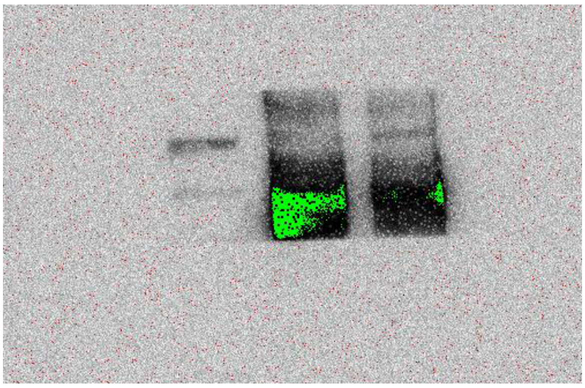

Fig.6. F. RBE IP: WWP1 WWP1

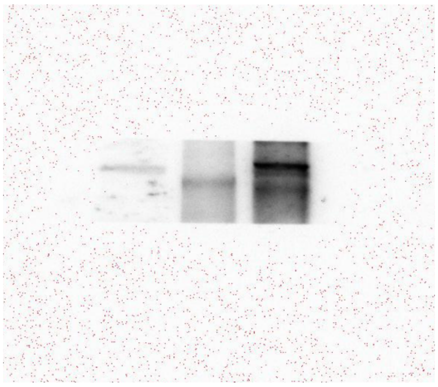

Fig.6. F. RBE IP: WWP1 NDFIP1

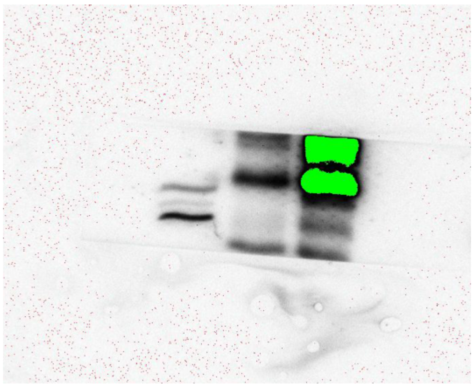

Fig.6. F. RBE IP: NDFIP1 NDFIP1

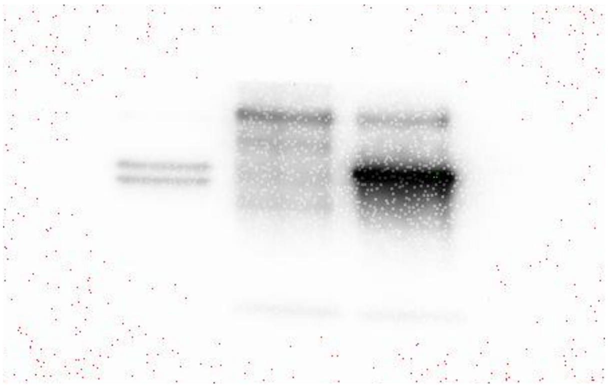

Fig.6. F. RBE IP: NDFIP1 WWP1

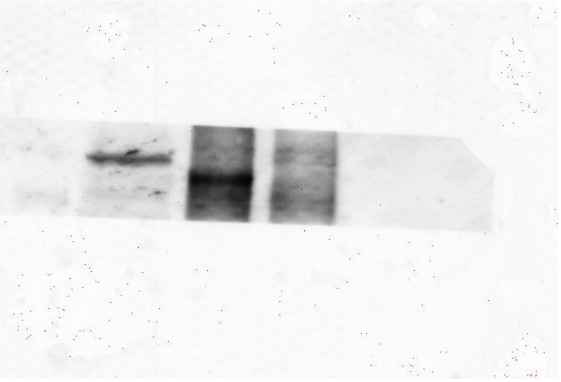

Fig.7

Fig.7. A. HuCCT1 WWP1

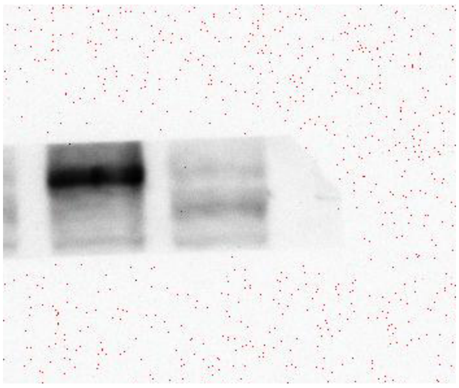

Fig.7. A. HuCCT1 NDFIP1

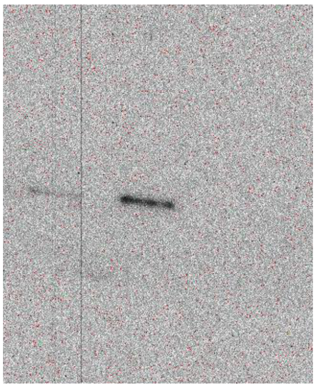

Fig.7. A. HuCCT1 GAPDH

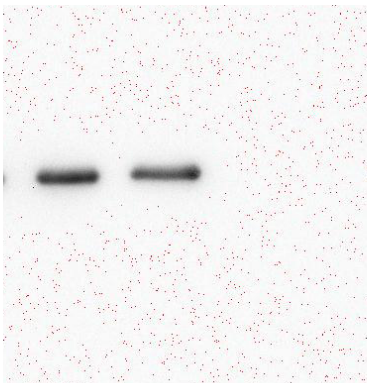

Fig.7. A. HCCC-9810 WWP1

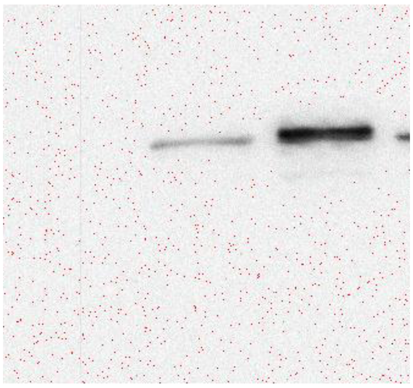

Fig.7. A. HCCC-9810 NDFIP1

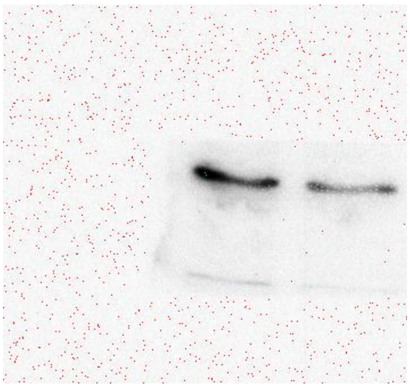

Fig.7. A. HCCC-9810 GAPDH

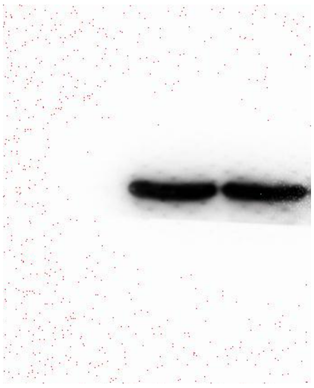

Fig.7. A. RBE WWP1

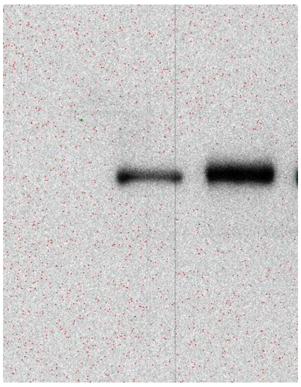

Fig.7. A. RBE NDFIP1

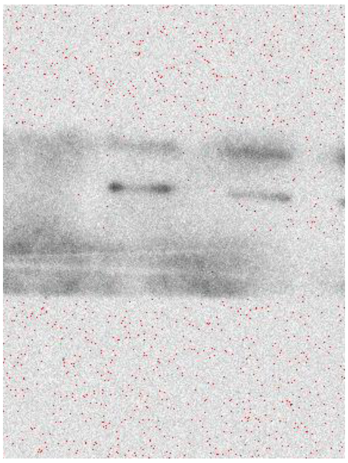

Fig.7. A. RBE GAPDH

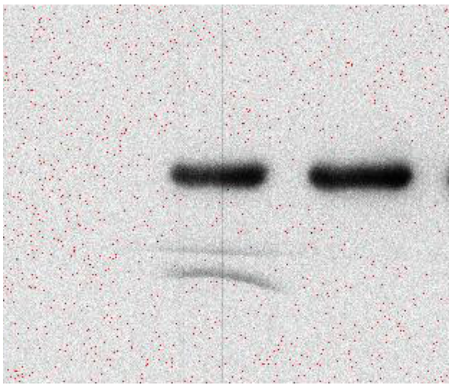

Fig.7. C.　WWP1

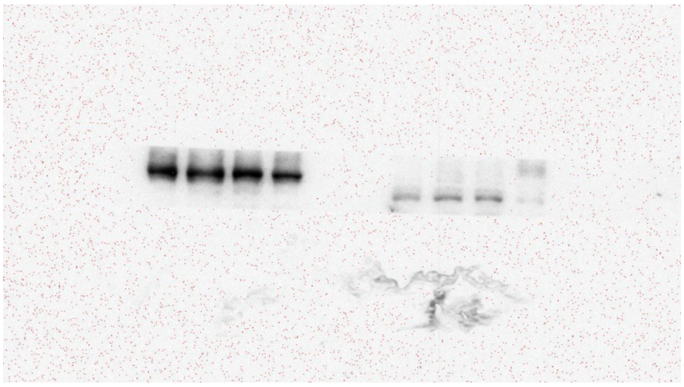

Fig.7. C.　NDFIP1

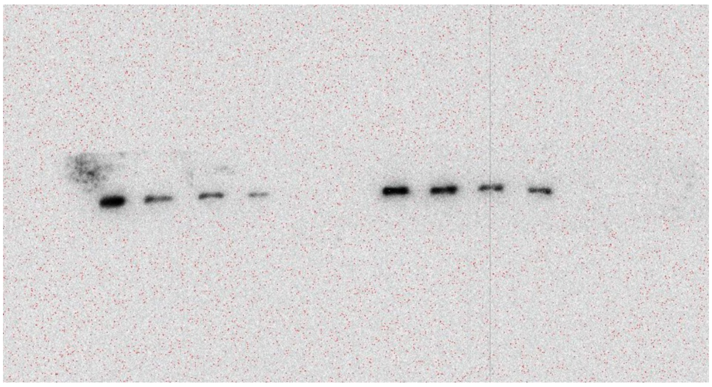

Fig.7. C.　GAPDH

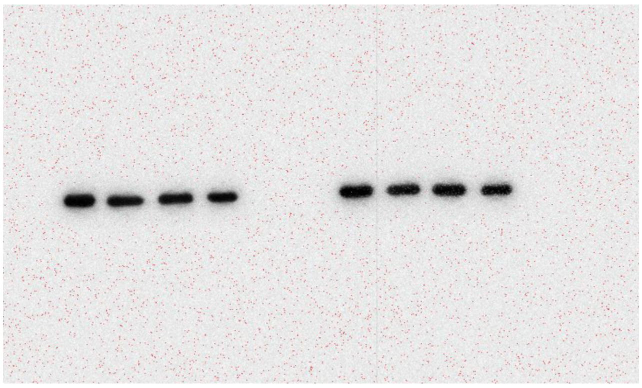

Fig.7. D. HCCC-9810 WWP1

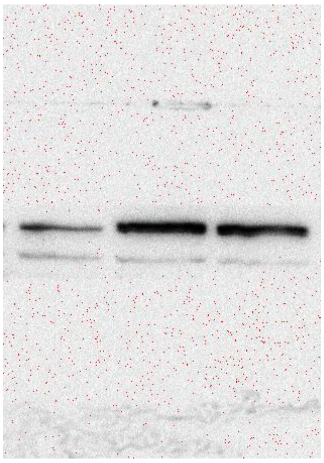

Fig.7. D. HCCC-9810 NDFIP1

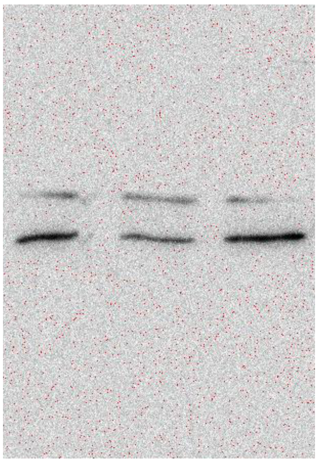

Fig.7. D. HCCC-9810 GAPDH

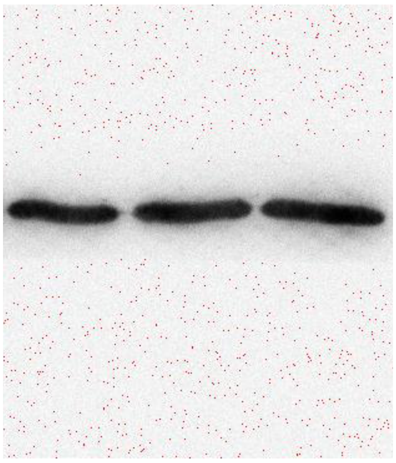

Fig.7. D. RBE WWP1

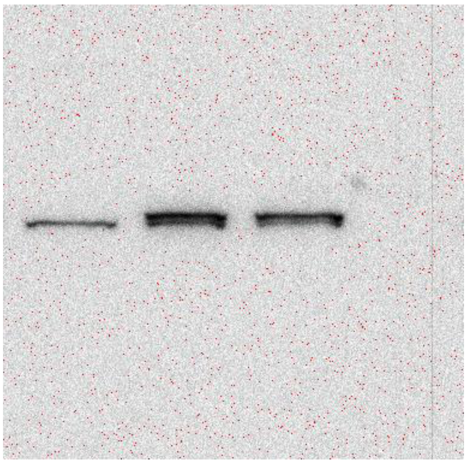

Fig.7. D. RBE NDFIP1

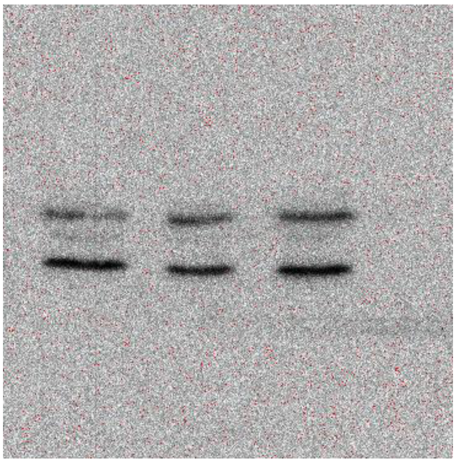

Fig.7. D. RBE GAPDH

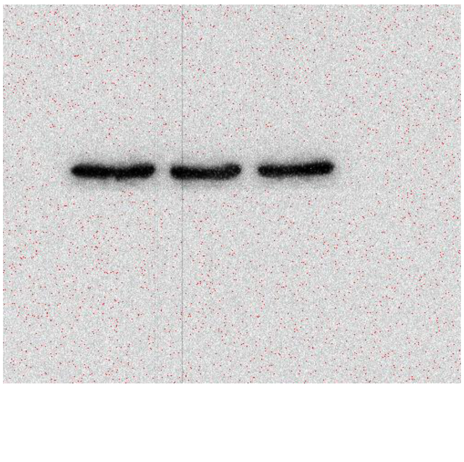

Fig.7. E. HuCCT1 Ubiquitin

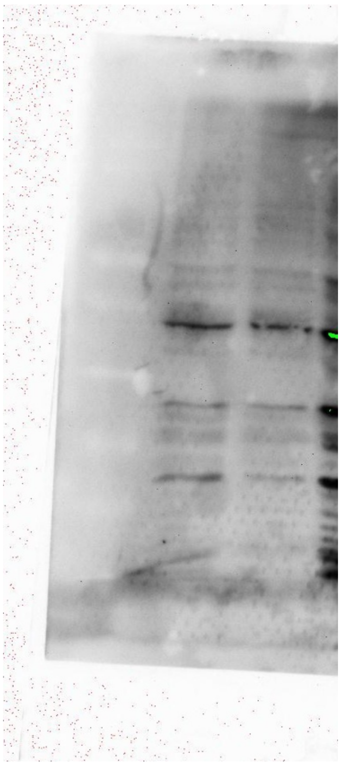

Fig.7. E. HuCCT1 WWP1

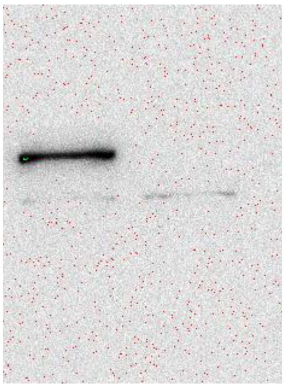

Fig.7. E. HuCCT1 NDFIP1

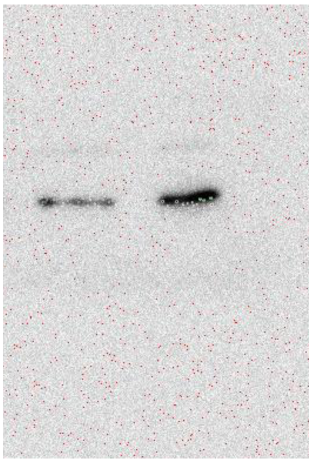

Fig.7. E. HuCCT1 GAPDH

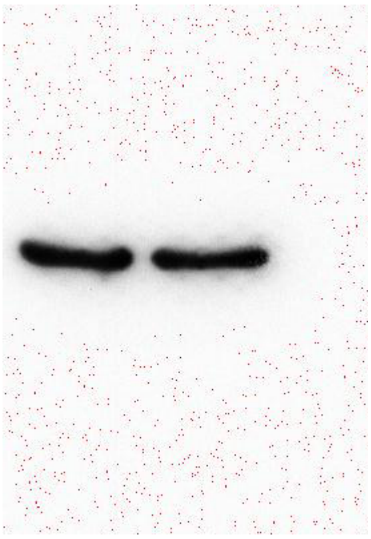

Fig.7. E. HCCC-9810 Ubiquitin

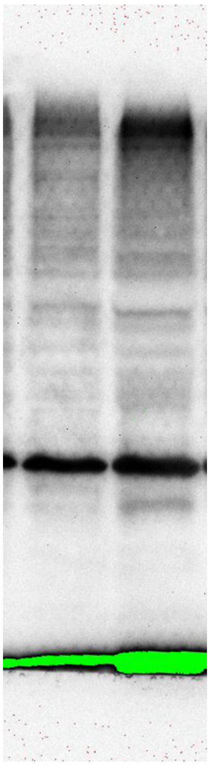

Fig.7. E. HCCC-9810 WWP1

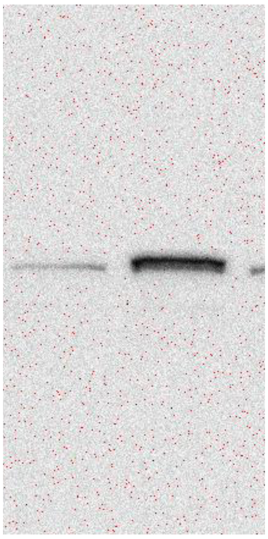

Fig.7. E. HCCC-9810 NDFIP1

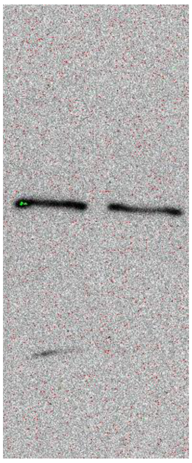

Fig.7. E. HCCC-9810 GAPDH

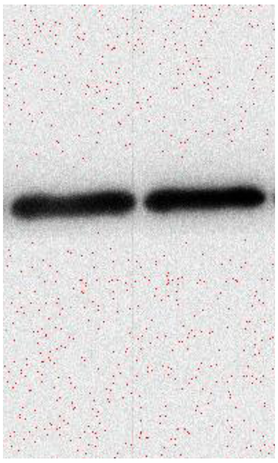

Fig.7. E. RBE Ubiquitin

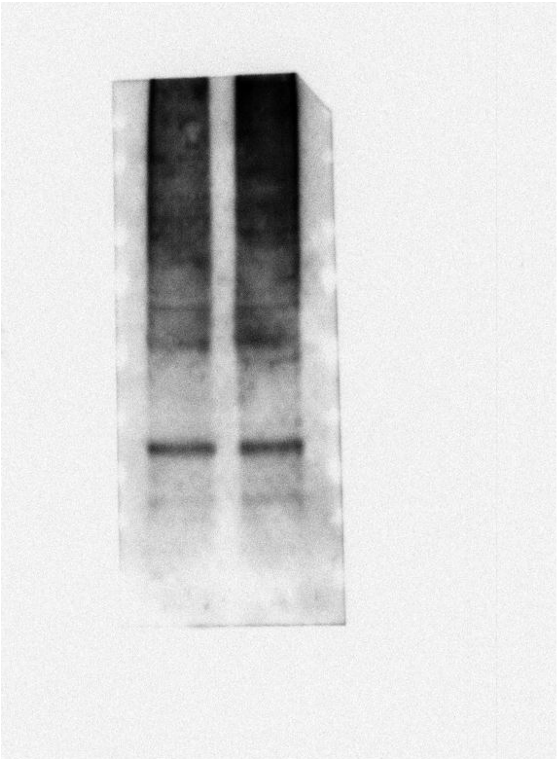

Fig.7. E. RBE WWP1

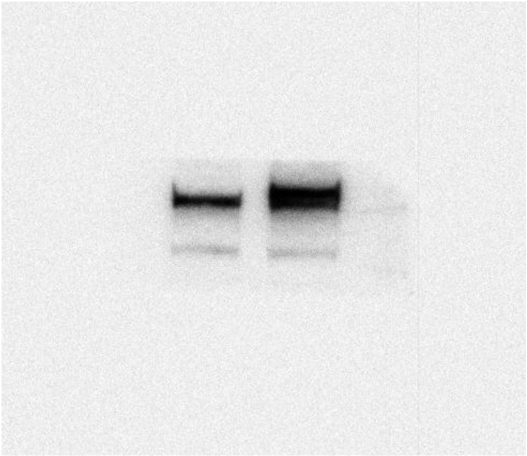

Fig.7. E. RBE NDFIP1

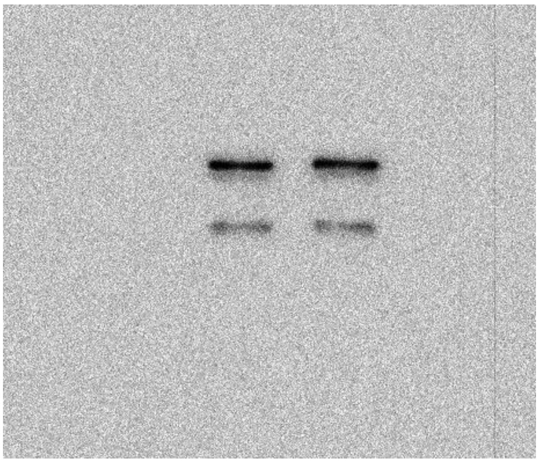

Fig.7. E. RBE GAPDH

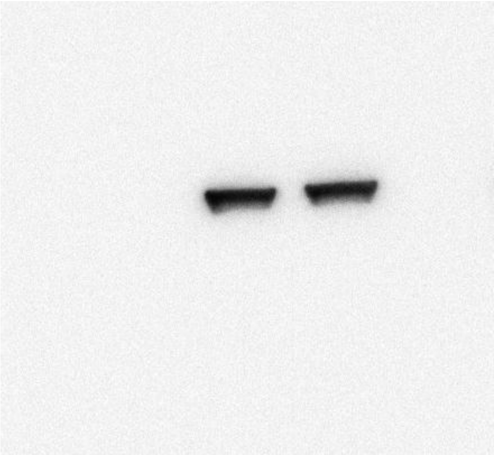

Fig.7. F. 293T MYC-Ubiquitin

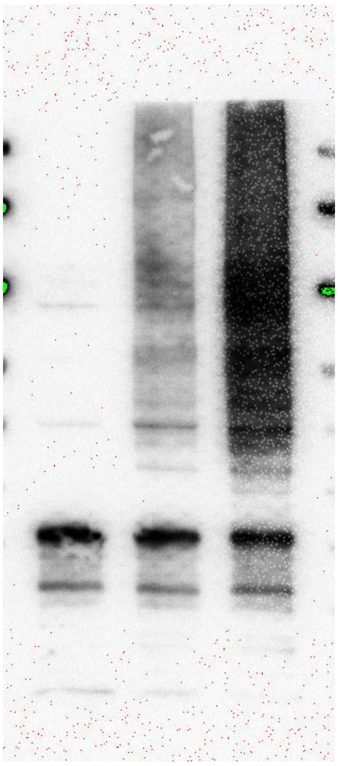

Fig.7. F. 293T HA-WWP1

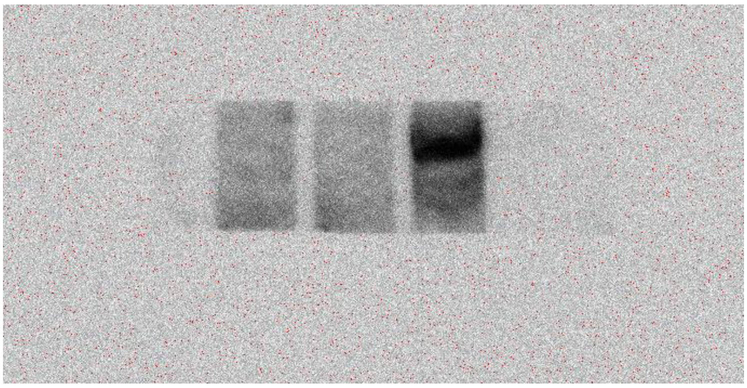

Fig.7. F. 293T Flag-NDFIP1

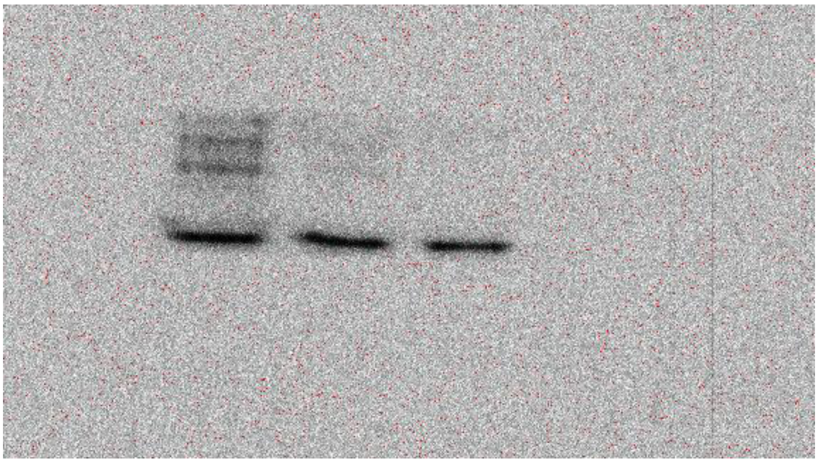

Fig.7. F. 293T GAPDH

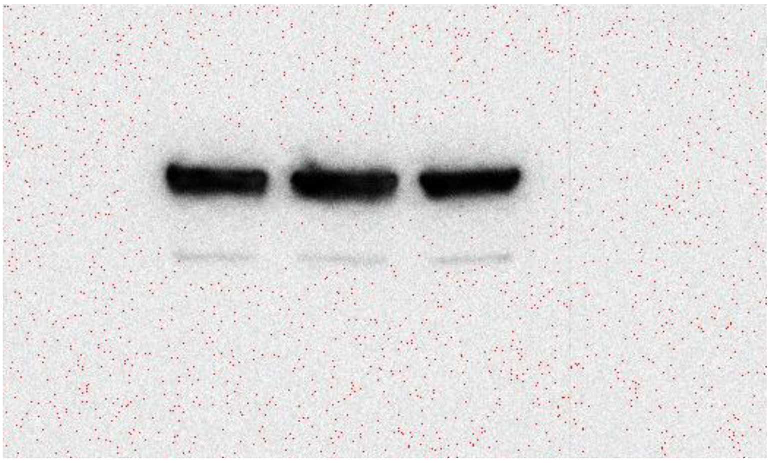

Fig.8

Fig.8. A. WWP1

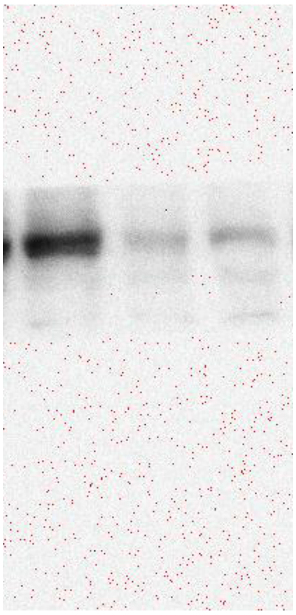

Fig.8. A. NDFIP1.

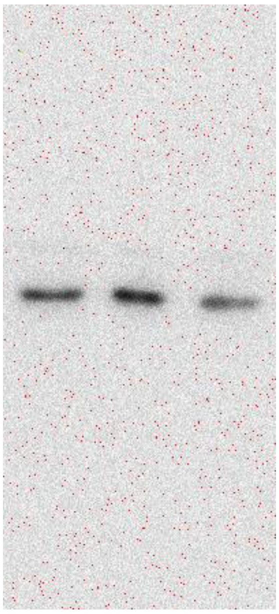

Fig.8. A. GAPDH.

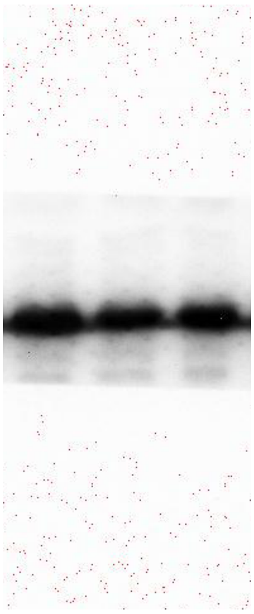

Fig.8. F CaseA. WWP1.

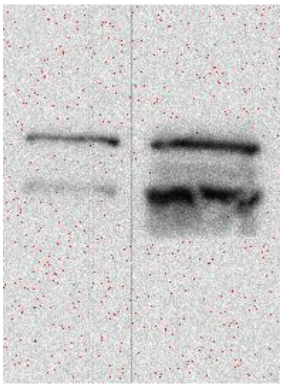

Fig.8. F CaseA. NDFIP1.

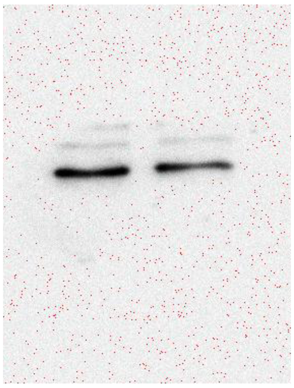

Fig.8. F CaseA.GAPDH

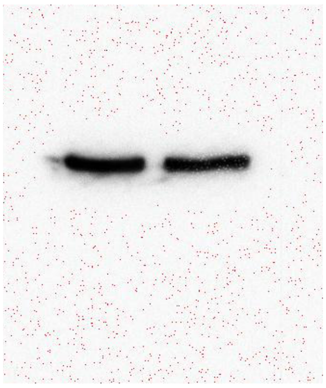

Fig.8.F CaseB. WWP1.

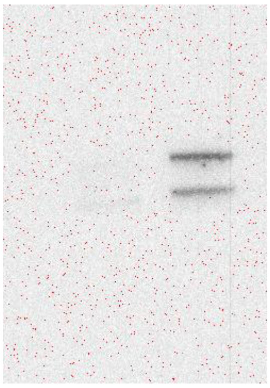

Fig.8.F CaseB. NDFIP1.

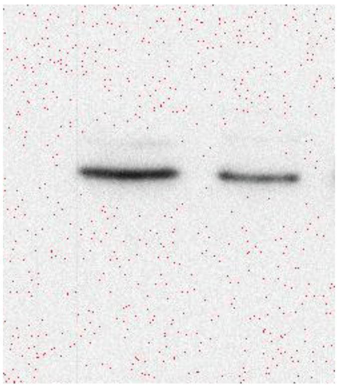

Fig.8.F CaseB.GAPDH

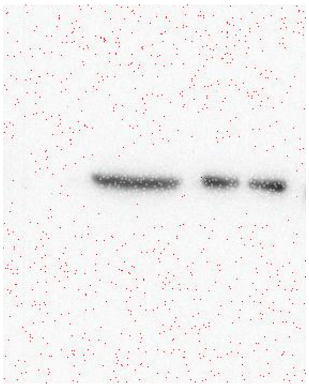

Fig.8.F CaseC. WWP1.

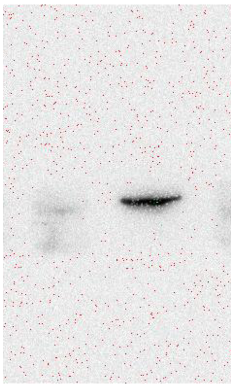

Fig.8.F CaseC. NDFIP1.

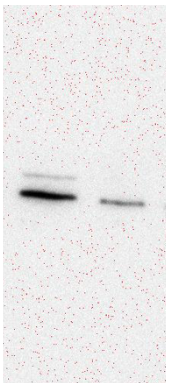

Fig.8.F CaseC.GAPDH

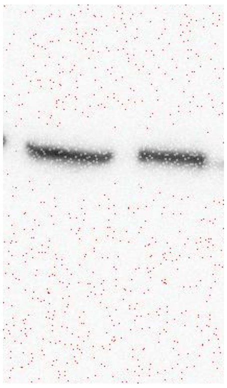

Fig.8.F CaseD. WWP1.

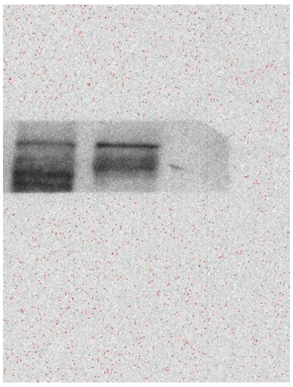

Fig.8.F CaseD. NDFIP1.

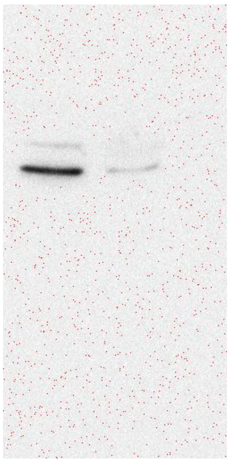

Fig.8.F CaseD.GAPDH

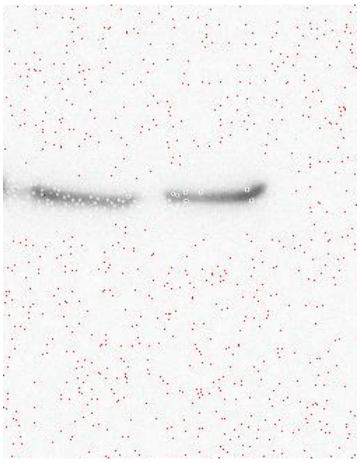

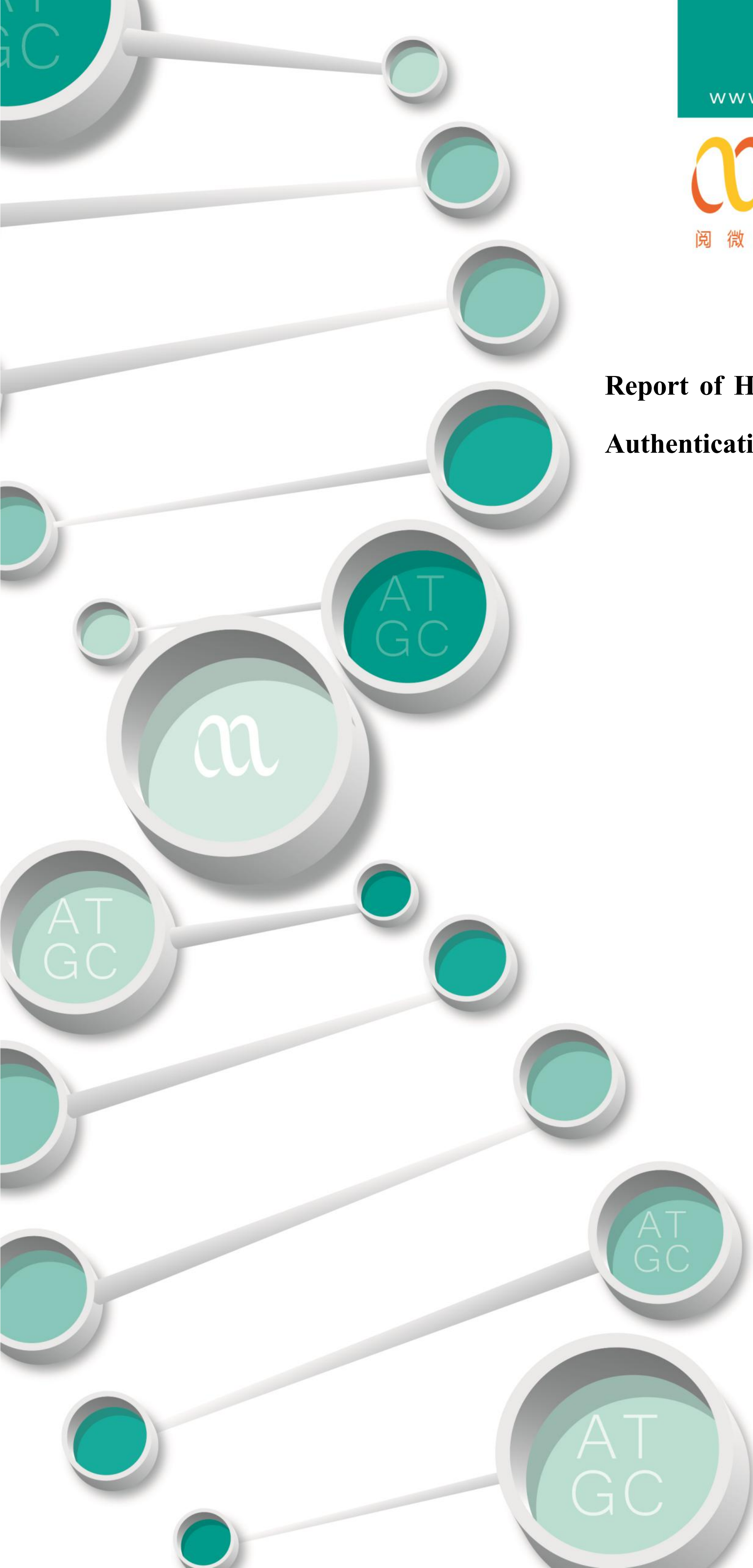

www.microread.com

阅微基因 Microread

阅 微 基 因　省 时 省 心

Report of Human Cell Line Authentication

Report of Human Cell Line Authentication

Delivery Date:2021-05-01

Analysis Date:2021-05-05

Ⅰ. Sample

Sample Name:'XB0421-0208', labeled as 'Li Yongjian 1', was received on 2021-05-01.

Ⅱ.Method and Procedure

Sample DNA was extracted by Microread Genomic DNA Kit.

PCR was amplified with STR Multi-amplification Kit (Microreader™21 ID System).

PCR products were assayed with ABI 3730xl DNA Analyzer (Applied Biosystems®).

Data were analyzed using GeneMapperID-X software and then compared with the ATCC, DSMZ and ExPASydatabases for reference matching.

Ⅲ. Results

1. The results of the negative and positive control match expectations.
2. The STR profiles of the test sample are clear and fine, which were shown in the attached figure1.
3. No loci of more than three alleles are observed in the 8 core STR loci[1] which is required to test in ANSI/ATCC ASN-0002-2011standard.
4. Results of database comparison[2] (the highest percent match to the query, please refer to the detailed information in Figure2, 3 and 4):

	ATCC	DSMZ	ExPASy
Cell Name	Calu-6Anaplastic	3A(tPA-30-1)	HCCC-9810

	CarcinomaHuman		
Cell No.	HTB-56	CRL-1583	CVCL_6908
Shared loci [3]	/	/	15
Matching[4]	89%	0.78	100%
Interpretation[5]	related	——	Identical

Remarks： 1. In the data comparison result, the 8 comparison genetic lociin the ANSI/ATCC ASN-0002-2011 standard are D5S818, D13S317, D7S820, D16S539, vWA, TH01, TPOX and CSF1PO. More than or equal to 3 multiple alleles in these 8 sitesindicates that there may be cross-contamination of homologous species;Less than 3 multiple alleles loci might result from trisomy or mutation.2.The genotypingresults of the detected cells are compared with ATCC and DSMZ databases (the DSMZ database contains the STR genotypes of 2455 cells from the 4 cell banks of ATCC, DSMZ, JCRB and RIKEN) and ExPASydatabases(Version 36 (October 2020)) (the ExPASydatabasecontains STR genotypesof 7,269 human cell lines from ATCC, DSMZ, JCRB, ECACC, Riken and other cell databases), cells that are not included in above databases will not be compared.3. The matching value of ATCC and ExPASy database is "Tanabe"2 x (number of alleles matching) / (total number of alleles in database profile and query sample) x 100%.The matchingvalue of DSMZ database is EV = (the number of generated peaks of test cell x2) / total number of peaks of (test cell + matched cell).4.The number of STR loci shared between the cells to be tested and the comparison result cells in the database.5. The interpretation of ATCC is based on "ANSI/ATCC ASN-0002-2011", giving "Identical" to matches =100%, "related" to matches that≥ 80%, "require further investigation" to matches that from 79% to 56%, and "unrelated" to those matches less than 56%. As comparing with DSMZ database, giving "Identical" to only when EV=1.

IV. Conclusions

Li Yongjian 1:

①No cross-contamination of other human cell line is found.

②The submitted profile has a 89% match for the following ATCC human cell line(s) in ATCC STR database (8 core loci plus Amelogenin):Calu-6Anaplastic CarcinomaHuman.

③ Between the submitted profile and 3A(tPA-30-1) ,the STR matching rate（EV value）is 0.78 in DSMZ STR database.

④The submitted profile has a 100% match on 15 STR of the following human cell line(s) in ExPASy database：HCCC-9810.

Operator: Ru You　　　　Auditor: Yingjie Sun

北京阅微基因技术股份有限公司 报告专用章

Beijing Microread Genetics Co., Ltd

Notes：

1. The short tandem repeat (STR) profile generated by Beijing Microread Genetics Co., Ltdis indicative only of the sample sent to Beijing Microread Genetics Co., Ltd at the time it was sent.
2. Beijing Microread Genetics performs STR Profiling following ISO 9001:2015 and ISO 13485:2016 quality standards.This data and analysis are for research use only.
3. Standards for Cell Line Authentications：To standardize STR analysis for human cell line authentication, the American Tissue Culture Collection (ATCC) Standards Development Organization Workgroup published ASN-0002-2011, which recommends the use of at least eight STR loci (TH01, TPOX, vWA, CSF1PO, D16S539, D7S820, D13S317 and D5S818) plus Amelogenin for gender identification for human cell line authentication.

Figure 1：STR profiles of Li Yongjian 1cell line

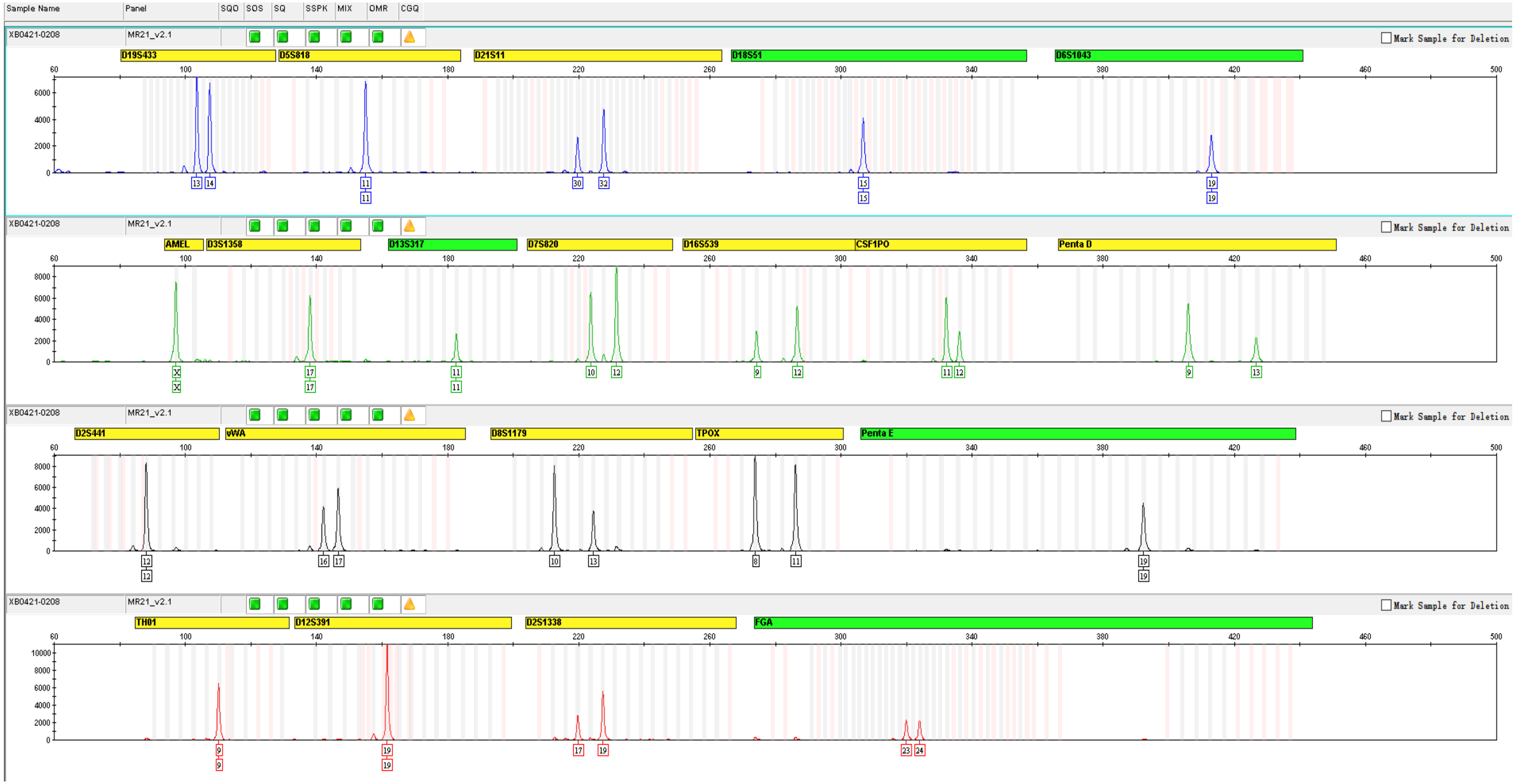

Figure2: Result of STR matching analysis in ATCC STR database

Add to Cart	%Match	ATCC® Number	Designation	D5S818	D13S317	D7S820	D16S539	vWA	TH01	AMEL	TPOX	CSF1PO
☐	89.0	HTB-56	Calu-6Anaplastic CarcinomaHuman	11	11	10	13	17	9	X	8	12
☐	82.0	HTB-126	Hs 578TBreast CarcinomaHuman	11	11	10	9,12	17	9,9.3	X	8	13
☐	80.0	CRL-1584	3A-sub EPlacentaHuman	11,13	11	10	9,12	16,17	9,9.3	X	8,11	10,12
☐	80.0	CRL-1583	3A(TPA-30-1)EpithelialHuman	11,13	11	10	9,12	16,17	9,9.3	X	8,11	10,12

Add to Cart Export to Excel

Disclaimer: Reference to this database and the data contained therein may be cited in publications, and ATCC encourages such citation or reference. While every reasonable effort has been made to assure the accuracy of these data, no warranty, express or implied, is made by ATCC as to their accuracy.

.

Figure3: Result of STR matching analysis in DSMZ STR database

Result of STR matching analysis by your data.

- DSMZ Profile Database -

A graphical presentation is shown at the bottom of this page.

EV	Cell No.	Cell name	D5S818	D13S317	D7S820	D16S539	VWA	TH01	AM	TPOX	CSF1PO	Figures
	Query (Your Cell)		11,11	11,11	10,12	9,12	16,17	9,9	X,X	8,11	11,12	
0.78(28/36)	CRL-1583	3A(tPA-30-1)	11,13	11,11	10,10	9,12	16,17	9,9.3	X,X	8,11	10,12	-
0.78(28/36)	CRL-1584	3A-sub E [post crisis of 3A(tPA-30-1)]	11,13	11,11	10,10	9,12	16,17	9,9.3	X,X	8,11	10,12	-
0.78(28/36)	CRL-1692	HISM	11,11	8,11	11,12	12,13	16,17	9,9.3	X,X	8,11	11,12	-
0.78(28/36)	RCB2531	CB-3512	11,11	8,11	10,11	9,14	16,17	9,9	X,X	8,11	10,12	-
0.72(26/36)	610	HAL-01	12,13	11,11	10,12	9,11	16,17	6,8	X,X	8,11	11,12	-
0.72(26/36)	679	BC-3	11,12	11,11	10,12	12,12	14,18	6,9	X,X	8,11	11,12	-
0.72(26/36)	707	HH	11,13	11,13	10,10	12,12	15,16	9,9	X,X	8,11	11,12	-
0.72(26/36)	CRL-2105	HH	11,13	11,12	10,10	12,12	15,16	9,9	X,X	8,11	11,12	-
0.72(26/36)	CRL-2277	BC-3	11,12	11,11	10,12	12,12	14,18	6,9	X,X	8,11	11,12	-
0.72(26/36)	HTB-142	Hs 602	11,11	11,12	12,12	8,10	16,17	9,9	X,X	8,11	11,11	-
0.72(26/36)	IFO50286	SF126	11,11	11,11	8,10	9,12	14,17	6,7	X,X	8,11	12,12	-
0.72(26/36)	IFO50311	SKG-IIIb	10,11	12,12	10,12	9,11	16,16	9,9	X,X	8,11	11,12	-
0.72(26/36)	JCRB0160	LI90	11,13	11,12	10,11	9,10	16,17	9,9	X,X	8,11	9,12	-
0.72(26/36)	JCRB1219	PL532	11,11	11,12	8,10	9,12	17,18	9,9	X,Y	8,11	12,12	-
0.72(26/36)	RCB0407	WS2TKB	11,12	12,14	10,12	9,12	17,18	7,9	X,X	8,11	11,12	-
0.72(26/36)	RCB0540	HAL-01	12,13	11,11	10,12	9,11	16,17	6,8	X,X	8,11	11,12	-
0.72(26/36)	RCB2263	HE47	11,11	11,12	10,12	9,11	17,17	6,9	X,X	11,11	11,12	-
0.70(28/40)	456	AC-1M81	11,11	9,11	10,12,13	12,13,14	14,16	9,10	X,X	8,11,12	11,12,10	-
0.67(24/36)	129	CX-1	11,12	11,11	10,10	11,12	17,19	6,9	X,X	8,9	11,12	-
0.67(26/39)	457	AC-1M88	10,11	9,11	10,12,13	12,13,14	14,16	9,10	X,X	8,11	11,12,10	-
0.67(24/36)	487	HCC-33	11,11	11,13	11,11	11,12	16,18	9,9	X,X	8,11	11,11	-

Figure 4： Result of STR matching analysis in ExPASy STR database

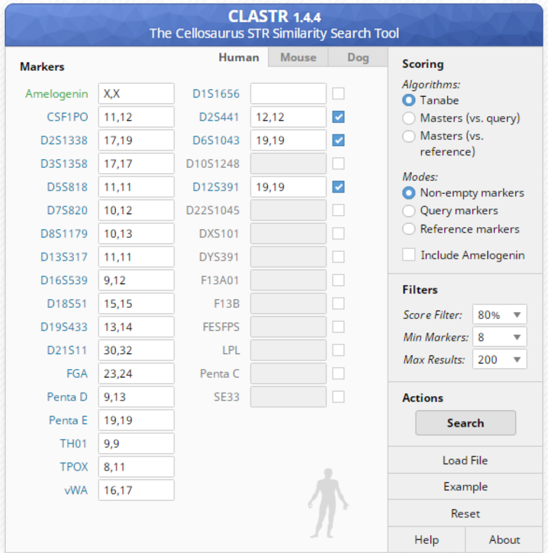

Results of similarity search on the 7440 human cell lines with STR profiles in Cellosaurus release 37.0

Export table

Accession	Name	Nº Markers	Score	Amel	CSF1PO	D2S1338	D2S441	D3S1358	D5S818	D6S1043	D7S820	D8S1179	D12S391	D13S317	D16S539	D18S51	D19S433	D21S11	FGA	Penta D	Penta E	TH01	TPOX	vWA
NA	Query	NA	NA	X,X	11,12	17,19	12,12	17,17	11,11	19,19	10,12	10,13	19,19	11,11	9,12	15,15	13,14	30,32	23,24	9,13	19,19	9,9	8,11	16,17
CVCL_6908	HCCC-9810	15	100.00%	X	11,12	17,19		17	11		10,12	10,13		11	9,12	15	13,14	30,32	23,24			9	8,11	16,17
CVCL_2820	3A(tPA-30-1)	8	81.48%	X	10,12				11,13		10			11	9,12							9,9.3	8,11	16,17

TOP

•

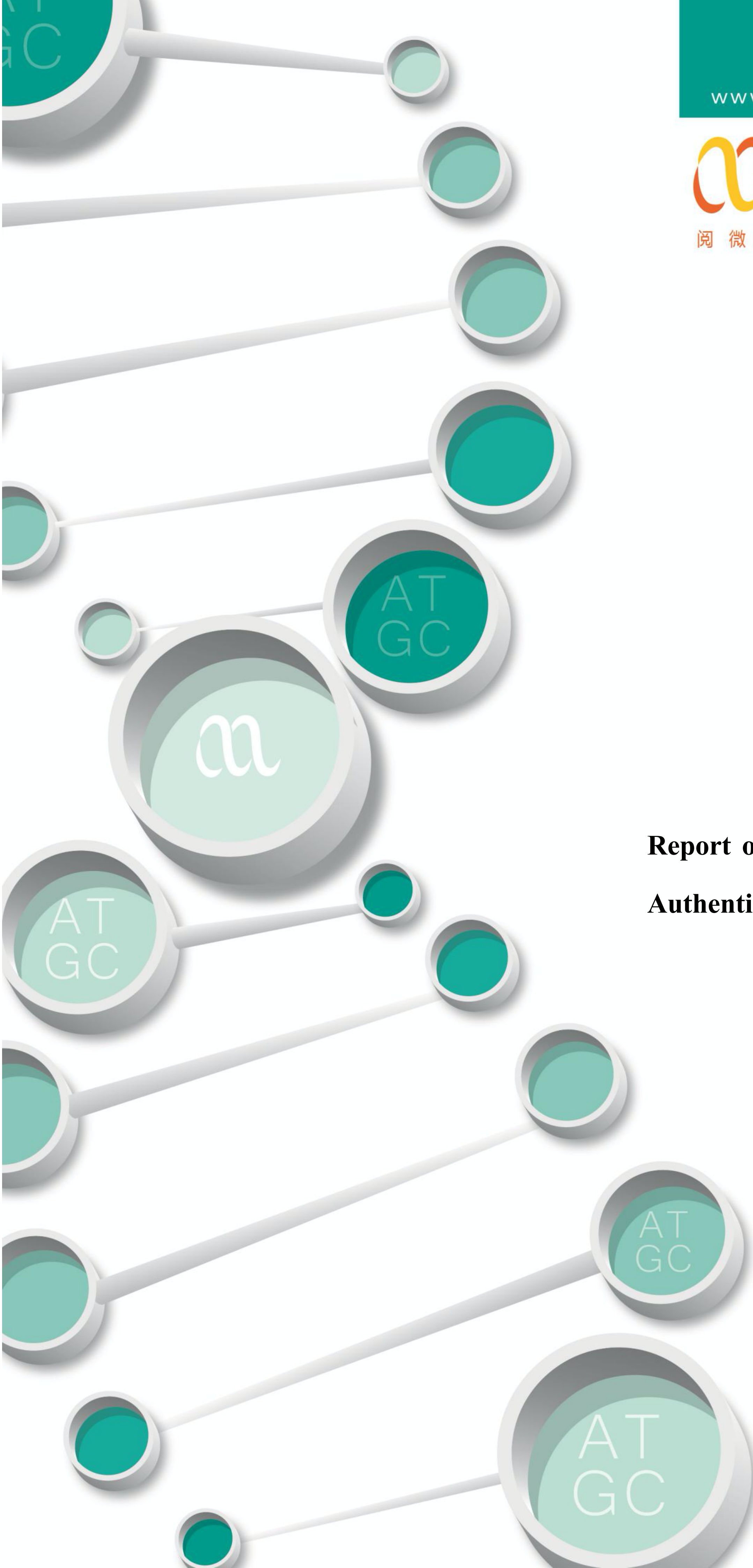

www.microread.com

阅 微 基 因　省 时 省 心

Report of Human Cell Line Authentication

Report of Human Cell Line Authentication

Delivery Date:2021-05-07

Analysis Date:2021-05-11

Ⅰ. Sample

Sample Name:‘XB0421-0251’, labeled as ‘Li Yongjian 3’, was received on 2021-05-07.

Ⅱ.Method and Procedure

Sample DNA was extracted by Microread Genomic DNA Kit.

PCR was amplified with STR Multi-amplification Kit (Microreader™21 ID System).

PCR products were assayed with ABI 3730xl DNA Analyzer(Applied Biosystems®).

Data were analyzed using GeneMapperID-X software and then compared with the ATCC and DSMZdatabases for reference matching.

Ⅲ. Results

The results of the negative and positive control match expectations.

The STR profiles of the test sample are clear and fine, which were shown in the attached table and figure.

Description of Multiple Alleles[1]:

Number of Multiple Alleles	Locus of Multiple Alleles
0	——

Results of database comparison[2] (the highest percent match to the query):

	ATCC	DSMZ	TestCell							
Cell Name	NCI-H2023Lung CarcinomaHuman	HuCCT1	Li Yongjian 3							
Cell No.	CRL-5912	JCRB0425	XB0421-0251							
Amelogenin	X	X,Y	X	Y						
D5S818	12	12,13	12	13						
D13S317	12	11,13	11	13						
D7S820	11	10,11	10	11						
D16S539	11,12	11,12	11	12						
vWA	18	18,18	18	18						
TH01	7,9	7,10	7	10						
TPOX	8	8,8	8	8						
CSF1PO	12	11,12	11	12						
Matching[3]	82%	1								
Interpretation[4]	related	Identical								

Remarks:

1. More than or equal to three peaks at more than three loci (multiple alleles loci)suggests there may be cross-contamination from homologous species. Less than 3 multiple alleles loci might result from trisomy or mutation.

2. The test results was comparedagainst STR DNA profiles recorded in ATCC and DSMZ(DSMZ database includes data sets of 2455 cell lines from ATCC, DSMZ, JCRB and RIKEN).

3. The matchingvalue of ATCC database is (the number of shared alleles between query sample and database profile) / （total number of alleles in database profile） x 100%. The matchingvalue of DSMZ database is EV = (the number of generated peaks of test cell x2) / total number of peaks of (test cell + matched cell).

4. The interpretation of ATCC is based on “ANSI/ATCC ASN-0002-2011”, giving “Identical” to matches =100%, “related” to matches that≥ 80%, “require further investigation” to matches that from 79% to 56%, and “unrelated” to those matches less than 56%.As comparing with DSMZ, giving “Identical” to only when EV=1.

IV. Conclusions

Li Yongjian 3:

①No cross-contamination of other human cell line is found.

②The submitted profile has a 82% match for the following ATCC human cell line(s) in ATCC STR database (8 core loci plus Amelogenin): NCI-H2023Lung CarcinomaHuman.

③ Between the submitted profile and HuCCT1 ,the STR matching rate（EV value）is1in DSMZ STR database.

Operator: Ru You　　　　Auditor: Xuejia Sun

北京阅微基因技术股份有限公司 报告专用章

Beijing Microread Genetics Co., Ltd

Notes：

1. The short tandem repeat (STR) profile generated by Beijing Microread Genetics Co., Ltdis indicative only of the sample sent to Beijing Microread Genetics Co., Ltd at the time it was sent.
2. Beijing Microread Genetics performs STR Profiling following ISO 9001:2015 and ISO 13485:2016 quality standards.
3. This data and analysis are for research use only.

Table: STR profiles of Li Yongjian 3 cell line

Cell line Li Yongjian 3（Fig.XB0421-0251）								
Marker	Allele 1	Allele 2	Allele 3	Allele 4	Allele 5	Allele 6	Allele 7	Allele 8
D19S433	13	13						
D5S818	12	13						
D21S11	31	31						
D18S51	13	13						
D6S1043	13	13						
AMEL	X	Y						
D3S1358	15	15						
D13S317	11	13						
D7S820	10	11						
D16S539	11	12						
CSF1PO	11	12						
PentaD	10	10						
D2S441	10	11						
vWA	18	18						
D8S1179	10	10						
TPOX	8	8						
PentaE	15	18						
TH01	7	10						
D12S391	18	20						
D2S1338	17	18						
FGA	20	23						

Figure1: STR profiles of Li Yongjian 3 cell line

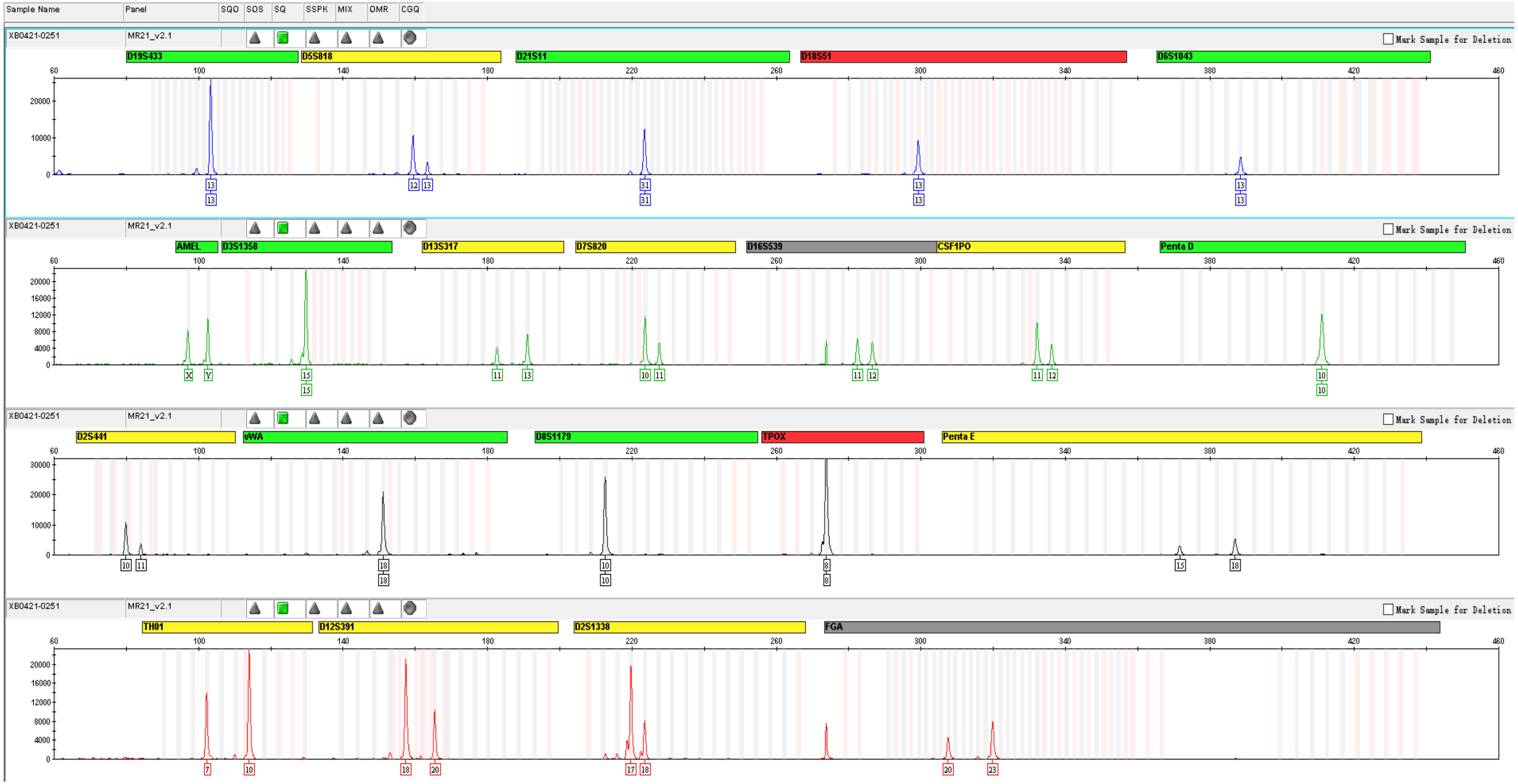

Figure2: Result of STR matching analysis in ATCC STR database

Add to Cart	%Match	ATCC® Number	Designation	D5S818	D13S317	D7S820	D16S539	vWA	TH01	AMEL	TPOX	CSF1PO
☐	82.0	CRL-5912	NCI-H2023Lung CarcinomaHuman	12	12	11	11,12	18	7,9	X	8	12
☐	80.0	CRL-1718	CCF-STTG1AstrocytomaHuman	12,13	11,13	10,11	11,12	17	7,8	X	8,11	12

Add to Cart | Export to Excel

Disclaimer: Reference to this database and the data contained therein may be cited in publications, and ATCC encourages such citation or reference. While every reasonable effort has been made to assure the accuracy of these data, no warranty, express or implied, is made by ATCC as to their accuracy.

While ATCC has largely used the Promega PowerPlex® 1.2 System in the creation of these data and recommends that researchers wishing to produce data for comparison also use a Promega PowerPlex® System ATCC does not provide a general endorsement of this product or provide any warranty or representation regarding its quality or performance in the scientific community for the identification of human cell lines.

Figure3: Result of STR matching analysis in DSMZ STR database

Result of STR matching analysis by your data.

- DSMZ Profile Database -

A graphical presentation is shown at the bottom of this page.

EV	Cell No.	Cell name	Locus names									Figures
			D5S818	D13S317	D7S820	D16S539	VWA	TH01	AM	TPOX	CSF1PO	
	Query (Your Cell)		12,13	11,13	10,11	11,12	18,18	7,10	X,Y	8,8	11,12	
1.00(36/36)	JCRB0425	HuCCT1	12,13	11,13	10,11	11,12	18,18	7,10	X,Y	8,8	11,12	-
1.00(36/36)	RCB1960	HuCCT1	12,13	11,13	10,11	11,12	18,18	7,10	X,Y	8,8	11,12	-
0.78(28/36)	CRL-2407	NK-92	12,13	9,12	10,11	11,12	18,18	6,9.3	X,Y	8,8	11,12	-
0.72(26/36)	488	NK-92	12,13	9,12	10,11	11,12	16,18	6,9.3	X,Y	8,8	11,12	-
0.72(26/36)	547	SKM-1	10,12	11,13	10,11	9,11	18,18	7,9	X,Y	8,11	10,12	-
0.72(26/36)	CRL-2408	NK-92MI	12,13	9,12	10,11	11,12	16,18	6,9.3	X,Y	8,8	11,12	-
0.72(26/36)	CRL-2409	NK-92CI	12,13	9,12	10,11	11,12	16,18	6,9.3	X,Y	8,8	11,12	-
0.67(24/36)	4	U-698-M	11,13	11,13	8,10	11,12	16,16	6,10	X,Y	8,8	12,12	-
0.67(24/36)	42	697	11,13	11,12	10,11	11,12	16,18	8,9	X,Y	8,11	11,12	-
0.67(24/36)	287	RPMI-2650	12,13	11,12	8,11	11,12	16,18	6,8	X,Y	8,8	9,11	-
0.67(24/36)	302	CAL-51	12,13	11,13	7,12	11,13	18,18	7,7	X,X	8,9	11,12	-
0.67(24/36)	CCL-30	RPMI 2650	12,13	11,12	8,11	11,12	16,18	6,8	X,Y	8,8	9,11	-
0.67(24/36)	CRL-1718	CCF-STTG1	12,13	11,13	10,11	11,12	17,17	7,8	X,X	8,11	12,12	-
0.67(24/36)	CRL-2885	sNF02.2	12,12	11,14	10,11	11,12	16,16	7,9	X,Y	8,12	11,12	-
0.67(24/36)	CRL-7802	Hs 454.T	11,12	8,13	8,10	11,12	17,19	6,10	X,Y	8,8	11,12	-
0.67(24/36)	JCRB0118	SKM-1	10,12	11,13	10,11	9,11	18,18	7,9	X,X	8,11	10,12	-
0.67(24/36)	JCRB9058	RPMI2650	12,13	11,12	8,11	11,12	16,18	6,8	X,Y	8,8	9,11	-
0.67(24/36)	RCB1977	CCF-STTG1	12,13	11,13	10,11	11,12	17,17	7,8	X,X	8,11	12,12	-
0.65(26/40)	RCB1936	MOLT-4F	11,12,13	12,13	7,8,10	11,12,13	17,18,19	6,8	X,Y	8,8	11,12	-
0.65(24/37)	84	MOLT-3	12,13	12,13	8,10,7	11,14	17,18	6,8	X,Y	8,8	11,12	-
0.63(26/41)	HB-8636	VLN3G2	11,12,13	9,11,13	9,10,11	11,12,13	16,17,20	8,9.3	X,Y	8,11	11,12	-

www.microread.com

阅微基因 Microread

阅 微 基 因　省 时 省 心

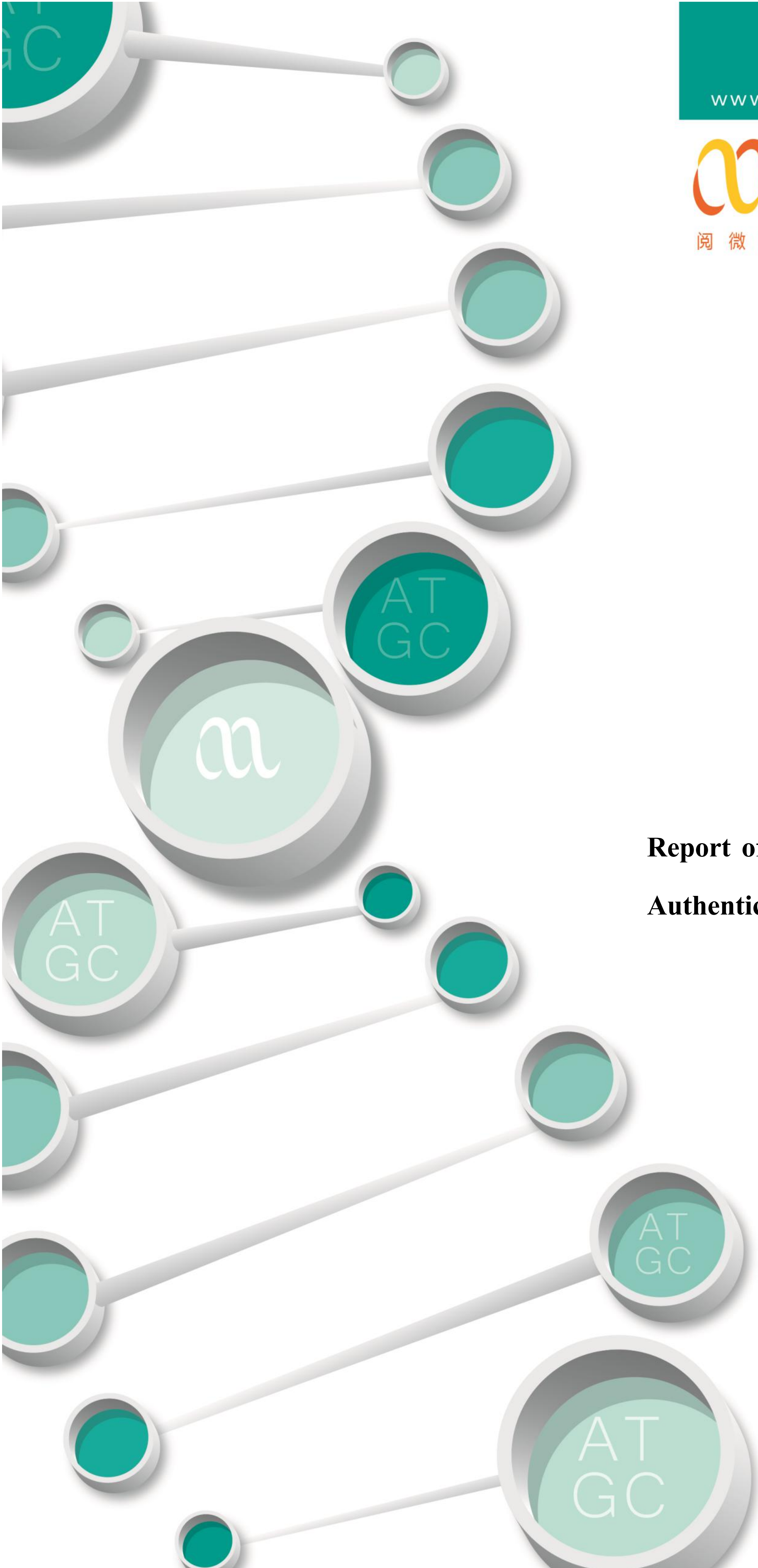

Report of Human Cell Line Authentication

Report of Human Cell Line Authentication

Delivery Date:2021-06-02

Analysis Date:2021-06-05

Ⅰ. Sample

Sample Name:'XB0421-0472', labeled as 'PKUPH-GDWK-2', was received on 2021-06-02.

Ⅱ.Method and Procedure

Sample DNA was extracted by Microread Genomic DNA Kit.

PCR was amplified with STR Multi-amplification Kit(Microreader^{TM}21 ID System).

PCR products were assayed with ABI 3730xl DNA Analyzer(Applied Biosystems®).

Data were analyzed using GeneMapperID-X software and then compared with the ATCC and DSMZdatabases for reference matching.

Ⅲ. Results

The results of the negative and positive control match expectations.

The STR profiles of the test sample are clear and fine, which were shown in the attached table and figure.

Description of Multiple Alleles[1]:

Number of Multiple Alleles	Locus of Multiple Alleles
0	——

Results of database compariso[n2] (the highest percent match to the query):

	ATCC	DSMZ	TestCell							
Cell Name	HCC1187Breast Ductal CarcinomaHuman	RBE	PKUPH-GDWK-2							
Cell No.	CRL-2322	RCB1292	XB0421-0472							
Amelogenin	X	X,X	X	X						
D5S818	12	11,12	11	12						
D13S317	11	9,9	9	9						
D7S820	8,11	8,11	8	11						
D16S539	10	10,11	10	11						
vWA	19	20,20	20	20						
TH01	6	6,6	6	6						
TPOX	8	8,8	8	8						
CSF1PO	13	12,12	12	12						
Matching[3]	70%	1								
Interpretation[4]	require further investigation	Identical								

Remarks:

1. More than or equal to three peaks at more than three loci (multiple alleles loci)suggests there may be cross-contamination from homologous species. Less than 3 multiple alleles loci might result from trisomy or mutation.

2. The test results was comparedagainst STR DNA profiles recorded in ATCC and DSMZ(DSMZ database includes data sets of 2455 cell lines from ATCC, DSMZ, JCRB and RIKEN).

3. The matchingvalue of ATCC database is (the number of shared alleles between query sample and database profile) / （total number of alleles in database profile）x 100%. The matchingvalue of DSMZ database is EV = (the number of generated peaks of test cell x2) / total number of peaks of (test cell + matched cell).

4. The interpretation of ATCC is based on “ANSI/ATCC ASN-0002-2011”, giving “Identical” to matches =100%, “related” to matches that≥ 80%, “require further investigation” to matches that from 79% to 56%, and “unrelated” to those matches less than 56%.As comparing with DSMZ, giving “Identical” to only when EV=1.

IV. Conclusions

PKUPH-GDWK-2:

①No cross-contamination of other human cell line is found.

②The submitted profile has a70% match for the following ATCC human cell line(s) in ATCC STR database (8 core loci plus Amelogenin): HCC1187Breast Ductal CarcinomaHuman.

③ Between the submitted profile and RBE ,the STR matching rate（EV value）is1in DSMZ STR database.

Operator: Ru You　　　　Auditor: Xuejia Sun

北京阅微基因技术股份有限公司 报告专用章

Beijing Microread Genetics Co., Ltd

Notes：

1. The short tandem repeat (STR) profile generated by Beijing Microread Genetics Co., Ltdis indicative only of the sample sent to Beijing Microread Genetics Co., Ltd at the time it was sent.
2. Beijing Microread Genetics performs STR Profiling following ISO 9001:2015 and ISO 13485:2016 quality standards.
3. This data and analysis are for research use only.

Table: STR profiles ofPKUPH-GDWK-2cell line

Cell linePKUPH-GDWK-2（Fig.XB0421-0472）								
Marker	Allele 1	Allele 2	Allele 3	Allele 4	Allele 5	Allele 6	Allele 7	Allele 8
D19S433	14	15.2						
D5S818	11	12						
D21S11	29	29						
D18S51	13	15						
D6S1043	14	14						
AMEL	X	X						
D3S1358	14	15						
D13S317	9	9						
D7S820	8	11						
D16S539	10	11						
CSF1PO	12	12						
PentaD	12	12						
D2S441	10	11						
vWA	20	20						
D8S1179	13	14						
TPOX	8	8						
PentaE	18	18						
TH01	6	6						
D12S391	18	18						
D2S1338	17	24						
FGA	19	24						

Figure1: STR profiles ofPKUPH-GDWK-2cell line

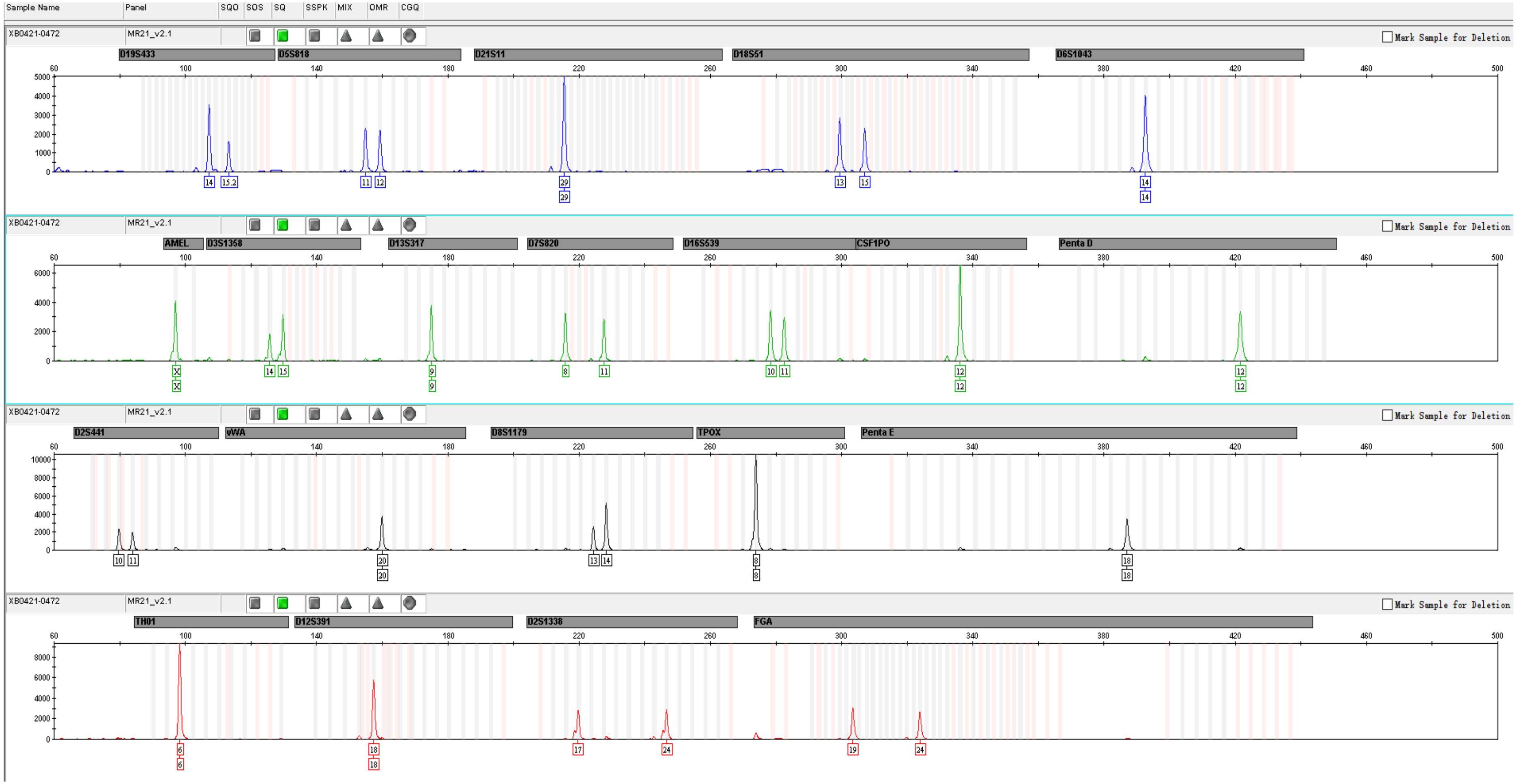

Figure2: Result of STR matching analysis in ATCC STR database

Add to Cart	%Match	ATCC® Number	Designation	D5S818	D13S317	D7S820	D16S539	vWA	TH01	AMEL	TPOX	CSF1PO
	70.0	CRL-2322	HCC1187Breast Ductal CarcinomaHuman	12	11	8,11	10	19	6	X	8	13
	69.0	CRL-2323	HCC1187 BLEBV-TransformedLymphoblast; Human	11,12	11	8,11	10,11	15,19	6	X	8	13
	64.0	HTB-34	MS751Cervical CarcinomaHuman	12	12	9,11	11	16	6	X	8	11,12
	64.0	crl-3127	UACC-1179Breast AdenocarcinomaHuman	12	12	8,11	10,13	16	6	X	8	10
	60.0	CRL-7573	Hs 840.TPharynx PapillomaHuman	11,12	12	8,11	11,13	15	6	X,Y	8,9	11,12
	60.0	CRL-2830	WT 9-7KidneyHuman	11,12	8,11	8,11	11,12	19	6,9.3	X	8	11,12
	60.0	CRL-2833	WT 9-12KidneyHuman	11,12	8,11	8,11	11,12	19	6,9.3	X	8	11,12
	60.0	CRL-2868	HCC827Lung CarcinomaHuman	12	9	11,12	12	18	6	X	8	11
	60.0	CRL-4013	CuFi-1Bronchial EpitheliumHuman	12	11,13	8,11	11,14	17,20	6,9.3	X	8,11	12
	59.0	CRL-8752	9D10B. LymphoblastHuman	11,12	9,13	8,11	11,13	14,17	6,7	X,Y	8	10,12

Figure3: Result of STR matching analysis in DSMZ STR database

Result of STR matching analysis by your data.

- DSMZ Profile Database -

A graphical presentation is shown at the bottom of this page.

EV	Cell No.	Cell name	Locus names									Figures
			D5S818	D13S317	D7S820	D16S539	VWA	TH01	AM	TPOX	CSF1PO	
	Query (Your Cell)		11,12	9,9	8,11	10,11	20,20	6,6	X,X	8,8	12,12	
1.00(36/36)	RCB1292	RBE	11,12	9,9	8,11	10,11	20,20	6,6	X,X	8,8	12,12	-
0.67(24/36)	449	CAL-78	11,12	8,12	11,11	8,11	16,16	6,6	X,X	8,8	12,12	-
0.67(24/36)	675	LS	12,13	9,11	8,10	10,11	14,16	6,8	X,X	8,8	12,12	-
0.67(24/36)	CRL-2323	HCC 1187BL	11,12	11,11	8,11	10,11	15,19	6,6	X,X	8,8	13,13	-
0.67(24/36)	CRL-2831	TS 9-7	11,12	8,11	8,11	11,12	19,19	6,6	X,X	8,8	11,12	-
0.67(24/36)	JCRB1200	NCC-RbC-53	11,12	9,9	8,11	10,10	18,18	9,9	X,X	8,8	11,12	-
0.67(24/36)	RCB0978	HIRS-BM	11,13	9,11	8,11	9,11	17,17	6,6	X,X	8,11	12,12	-
0.61(22/36)	133	8305C	10,13	9,9	8,8	10,11	14,16	6,7	X,X	8,8	9,12	-
0.61(22/36)	430	SK-MM-2	9,11	10,10	8,11	11,11	14,20	6,6	X,X	8,11	10,12	-
0.61(22/36)	541	JJN-3	11,12	9,9	8,9	11,12	15,15	9,9	X,X	8,10	12,12	-
0.61(22/36)	696	OAC-M5.1	11,12	11,12	8,8	10,11	19,20	6,9.3	X,X	8,8	10,13	-
0.61(22/36)	CRL-2830	WT 9-7	11,12	8,11	8,11	11,12	19,19	6,9.3	X,X	8,8	11,12	-
0.61(22/36)	CRL-2832	WT 9-9	11,12	8,11	8,11	11,12	19,19	6,9.3	X,X	8,8	11,12	-
0.61(22/36)	CRL-2833	WT 9-12	11,12	8,11	8,11	11,12	19,19	6,9.3	X,X	8,8	11,12	-
0.61(22/36)	CRL-4013	CuFi-1	12,12	11,13	8,11	11,14	17,20	6,9.3	X,X	8,11	12,12	-
0.61(22/36)	CRL-7166	Hs 204.Sp	11,11	8,9	10,11	11,13	15,16	6,6	X,X	8,8	11,12	-
0.61(22/36)	CRL-7178	Hs 216.We	12,13	9,9	7,10	10,11	15,16	8,8	X,X	8,8	12,12	-
0.61(22/36)	CRL-7364	Hs 605.Sk	11,13	9,12	10,11	10,11	15,18	6,9.3	X,X	8,8	12,14	-
0.61(22/36)	CRL-7365	Hs 605.T	11,13	9,12	10,11	10,11	15,18	6,9.3	X,X	8,8	12,14	-
0.61(22/36)	CRL-8752	9D10	11,12	9,13	8,11	11,13	14,17	6,7	X,Y	8,8	10,12	-
0.61(22/36)	JCRB0824	8305C	10,13	9,9	8,10	10,11	14,16	6,7	X,X	8,8	9,12	-

中国典型培养物保藏中心
CHINA CENTER FOR TYPE CULTURE COLLECTION (CCTCC)
Wuhan University, Wuhan 430072, China
Phone: 86-027-68752093 **Fax:** 86-027-68754833 **Email:** shenchao@whu.edu.cn

07-09-2021

Entrusted by Peking university people's hospital, CCTCC has conducted identification experiments on the HUH28 cell line, and came to the following conclusions:

1. There was only one forth allele found in D19S433 loci of HUH28 cell line, it indicating that there was no cross-contaminant of human source cell line.
2. Compared the STR data of HUH28 cell line in the databases of ATCC, DSMZ and CELLOSAURUS, its profile does not exactly match with any of the current data (Table 1).
3. The STR data of HUH28 cell line and HuH-28 (JCRB0426) cell line matches the rate of 96.30% in CELLOSAURUS database.

中国典型培养物保藏中心 业务专用章

Manager:

China Center for Type Culture Collection

Note:

1. The result is only responsible for the test sample, and the genomic DNA will be reserved for three month.
2. Reference of human cell line authentication: ANSI/ATCC ASN-0002-2011

Table1. The alleles of 21 loci in HUH28 cell line

HUH28 cell line (Fig. No. SJ-00874)				
Marker	Allele 1	Allele 2	Allele 3	Allele 4
D19S433	11.2	13	14.2	16.2
**D5S818**	**9**	**12**		
D21S11	30	33.2		
D18S51	15	15		
D6S1043	13	13		
**AMEL**	**X**	**X**		
D3S1358	15	16		
**D13S317**	**9**	**12**		
**D7S820**	**10**	**11**		
**D16S539**	**9**	**9**		
**CSF1PO**	**9**	**12**		
Penta D	9	10		
D2S441	12	12		
**vWA**	**17**	**17**		
D8S1179	11	11		
**TPOX**	**8**	**8**		
Penta E	13	22		
**TH01**	**9**	**9**		
D12S391	18	18		
D2S1338	19	19		
FGA	23	23		

CTCC

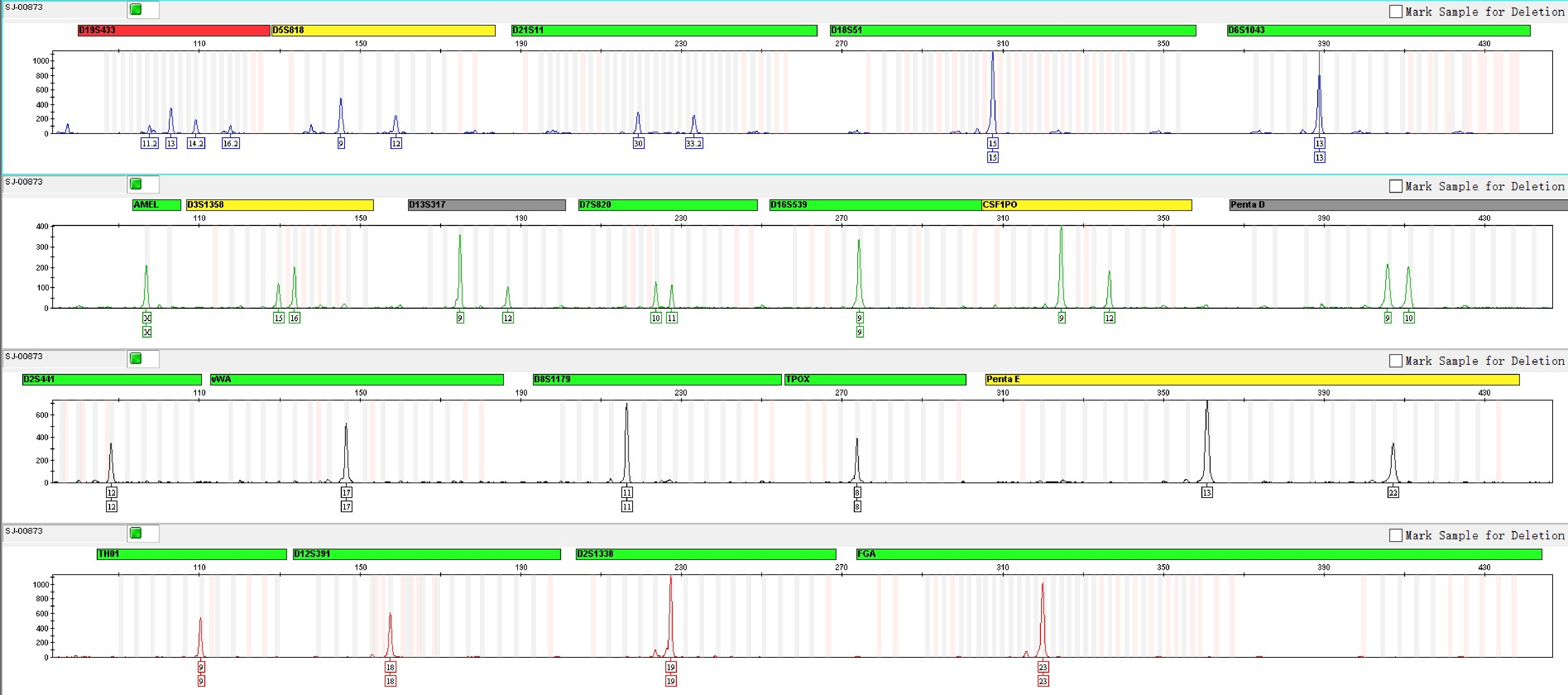
SJ-00873
Mark Sample for Deletion
D19S433 D5S818 D21S11 D18S51 D6S1043
11.2 13 14.2 16.2
9 12
30 33.2
15 15
13 13
SJ-00873
Mark Sample for Deletion
AMEL D3S1358 D13S317 D7S820 D16S539 CSF1PO Penta D
X X
15 16
9 12
10 11
9 9
9 12
9 10
SJ-00873
Mark Sample for Deletion
D2S441 vWA D8S1179 TPOX Penta E
12 12
17 17
11 11
8 8
13 22
SJ-00873
Mark Sample for Deletion
TH01 D12S391 D2S1338 FGA
9 9
18 18
19 19
23 23
