# Supplementary material for: WWP1 upregulation predicts poor prognosis and promotes tumor progression by regulating ubiquitination of NDFIP1 in intrahepatic cholangiocarcinoma
Source: Cell Death Discov. 2022 Mar 9;8:107. doi: 10.1038/s41420-022-00882-0 (PMC8906119; doi:10.1038/s41420-022-00882-0)
Supplement: Supplementary file 2 — Author Contributions Statement [file 41420_2022_882_MOESM2_ESM.docx]

**Author Contributions Statement**

Yongjian Li, Qian Cheng and Jiye Zhu designed the study and wrote the manuscript. Jie Gao, Zhuomiaoyu Chen and Chao Zhang collected the clinical samples. Jiaxi Zheng, Jingheng Guo and Zuyin Li performed experiments using clinical samples. Yongjian Li performed cell and animal experiments. Lingyu Tian and Yuzi Li provided technical and material support. Qian Cheng, Zhao Li and Jiye Zhu revised the manuscript.
